# Graphene-Oxide-Based Organic Solvent Filtration Membranes: Recent Progress and Perspectives

**DOI:** 10.1021/cbe.6c00026

**Published:** 2026-06-14

**Authors:** Jiwon Kim, Junhyeok Kang, Ju Yeon Kim, Wooyoung Choi, Taeyoung Kim, Huiseon Jang, Jeongyeob Oh, Younghoon Joung, Dae Woo Kim

**Affiliations:** Department of Chemical and Biomolecular Engineering, 26721 Yonsei University, Yonsei-ro 50, Seodaemun-gu, Seoul 03722, Republic of Korea

**Keywords:** graphene, membrane, organic solvent nanofiltration, purification, separation

## Abstract

Graphene-based membranes can be promising platforms for organic solvent nanofiltration (OSN) due to their unique two-dimensional nanochannels, chemical robustness, and tunable transport pathways. In particular, graphene oxide (GO) and nanoporous graphene architectures offer opportunities to overcome permeability/selectivity trade-off through controlled interlayer spacing, engineered nanopores, and tailored solvent–membrane interactions. This review provides a comprehensive overview of recent advances in multilayer graphene-based OSN membranes, focusing on synthesis strategies, transport mechanisms, and structural engineering approaches that enable high-performance separation. We discuss the synergistic roles of nanopore-mediated through-plane transport and confined interlayer diffusion, highlighting how solvent affinity, swelling behavior, and interfacial slip collectively determine separation performance. Recent progress in pore structure refinement, interlayer regulation, nanosheet orientation control, and aspect-ratio control is summarized to provide key design principles for achieving ultrafast permeance while maintaining sharp molecular separation. Beyond material design, emerging industrial applications, including purification, concentration, solvent exchange, pharmaceutical processing, electronic-grade solvent polishing, and organic solvent reverse osmosis, are broadly discussed. Finally, we checked remaining challenges toward scale-up and commercialization, including stability under harsh solvents, module compatibility, and realistic performance evaluation.

## Graphical abstract

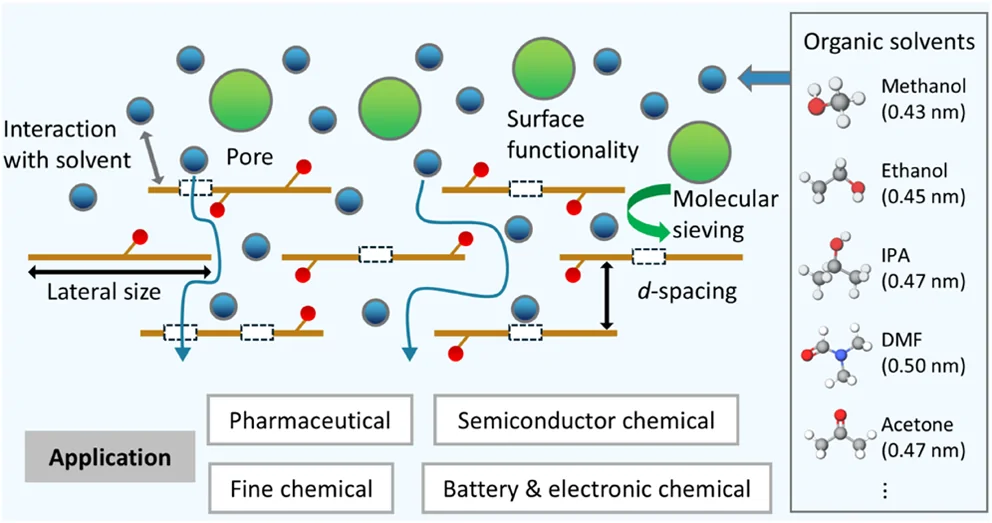

## 1 Introduction

Organic solvent-based processes have been widely used across the pharmaceutical, fine chemical, and electronic-material industries, where a wide range of organic solvents are routinely used to enhance product purity and process efficiency.^1–4^ However, as solvent usage increases, the energy demand and cost associated with solvent recovery and purification rise accordingly. Therefore, pressure is growing to reduce solvent waste and energy consumption in sustainable ways.^5^ Organic solvent nanofiltration (OSN) has attracted significant attention as a low-energy, pressure-driven membrane separation without phase change.^6,7^ Because of mild operation conditions at mild temperatures, OSN is readily integrated into continuous manufacturing, providing a practical route for high-value molecular separations, catalyst/additive recovery, and closed-loop solvent recycling.^7–9^

Among various types of graphene and two-dimensional materials, graphene oxide (GO)-based membranes are attractive for OSN because GO nanosheets assemble into lamellar structures with interlayer nanochannels that can act as molecular sieves (Figure 1A).^10^ Multiple independent design parameters, including the interlayer spacing, defects/pores, flake lateral size, and surface functionality, can be engineered simultaneously.^11–13^ OSN performance of GO laminates is not governed solely by an effective channel width. Rather, it reflects the combined effects of solvent-dependent interlayer swelling, chemical heterogeneity between oxidized and graphitic domains, and resistance/transport pathways introduced by defects and alignment.^14–17^ This multiparameter tunability makes GO a compelling material for ultrathin, selective layers on a chemically robust platform in organic media, complementing polymeric OSN membranes that can suffer from solvent-induced swelling or plasticization under harsh conditions with low permeance (Figure 1B).^6,18^ In order to realize this potential in a practical separation process, it is required to solve several interlinked issues. Permeance enhancement must be achieved by controlling pores/defects and laminate alignment of GO nanosheets, including ordered stacking and orientational uniformity. At the same time, the selective layer must remain mechanically stable on porous supports during long-term operation without delamination, and the nanochannels must be stabilized against swelling and structural compaction in strong solvents such as *N*-methyl-2-pyrrolidone (NMP) and dimethylformamide (DMF). These needs shift the research area from isolated performance records to engineering validation across module designs and pilot-scale durability.

**Figure 1:**
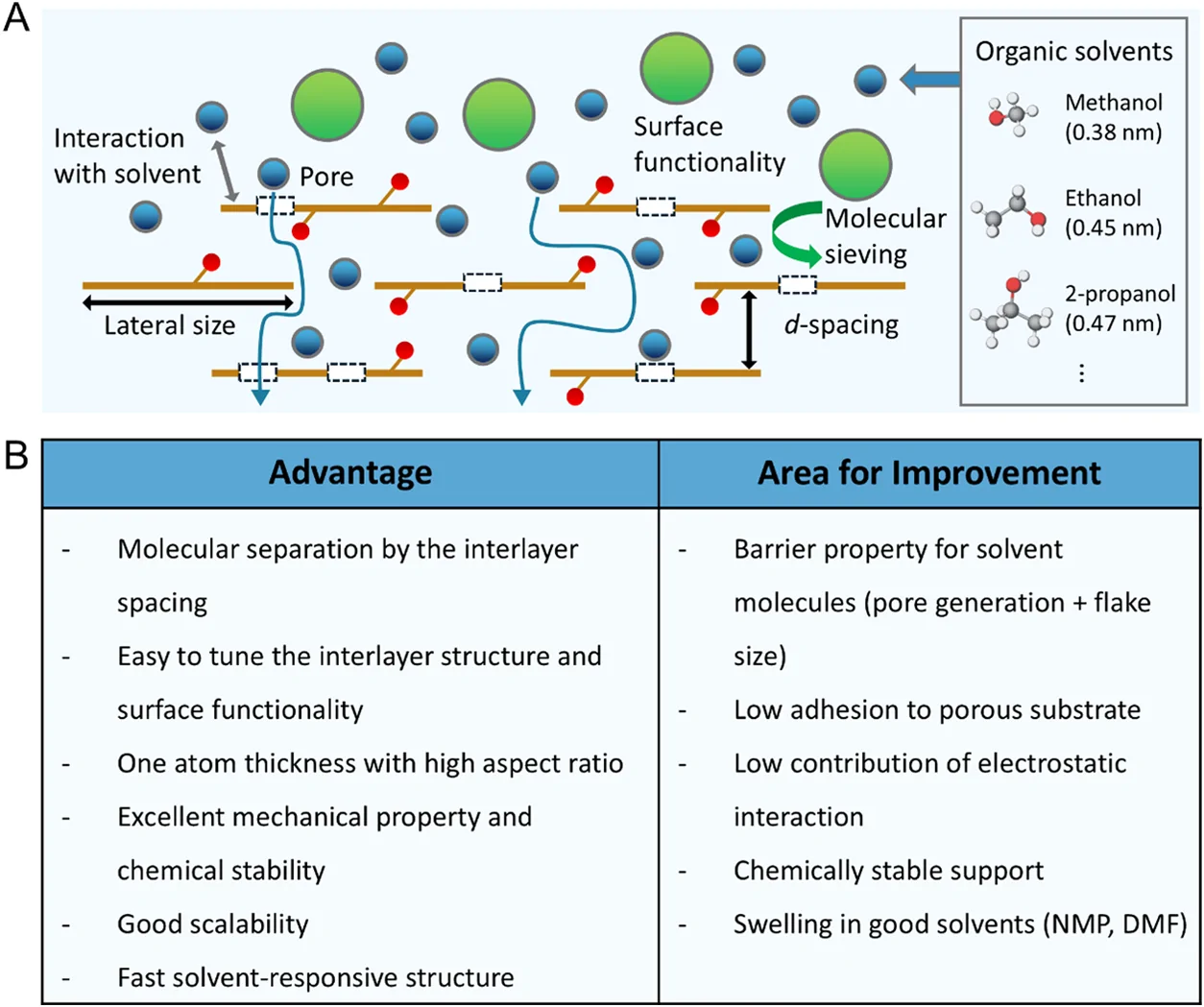
(A) Schematic illustration of organic solvent transport and separation mechanisms in graphene-based membranes. (B) Summary of the advantages and areas for improvement of graphene-based membranes for organic solvent transport and separation.

Based on a series of Web of Science topic searches over the past five years (2021–2025), OSN membrane research remains dominated by polymeric materials, especially polyamide (PA), yet graphene-based materials have attracted increasing attention as an alternative to OSN membranes (Figure 2A). In practice, graphene-based OSN studies are disproportionately concentrated on alcohols (Figure 2B). However, OSN solvents span a wide range of polarity, dielectric constant, viscosity, and molecular size, such that membrane performance can shift markedly with solvent (Table 1). This concentration in a narrow solvent window therefore emphasizes the need for broader, solvent-diverse investigations to establish generalizable structure–transport relationships and design rules for graphene-based OSN membranes. Meanwhile, OSN still represents a relatively small proportion of overall graphene-membrane research compared with water-treatment applications (Figure 2C). However, OSN remains comparatively underexplored in graphene-based membranes, which are still largely centered on water-treatment applications. Because dominant transport/separation behaviors and degradation mechanisms can differ fundamentally between water and organic solvents, water-based performance cannot be directly extended to OSN without OSN-specific design (Table 2). Nevertheless, on MWCO–permeance performance comparison, graphene-based membranes have reported superior OSN performance (Figure 2D–E).^19–43^ These results highlight the potential of graphene-based nanochannels as a distinct and tunable platform for OSN separations beyond water treatment.

**Figure 2:**
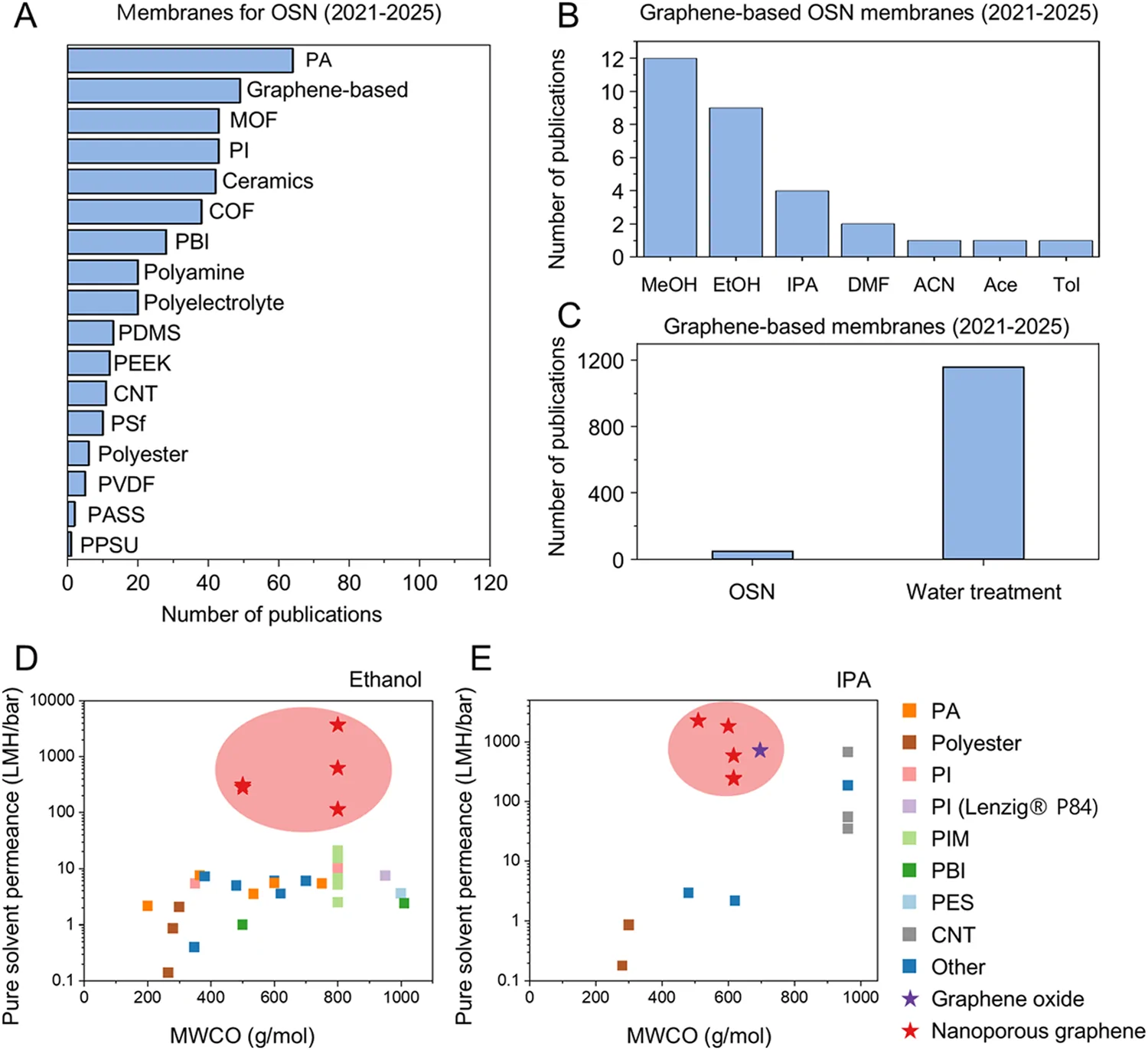
Publication trends from 2021 to 2025 based on topic searches in Web of Science. (A) Distribution of OSN membranes categorized by selective-layer materials (polyamide (PA), graphene-based, metal–organic framework (MOF), polyimide (PI), ceramics, covalent organic framework (COF), polybenzimidazole (PBI), polyamine, polyelectrolyte, polydimethylsiloxane (PDMS), poly­(ether ether ketone) (PEEK), carbon nanotube (CNT), polysulfone (PSf), polyester, poly­(vinylidene fluoride) (PVDF), poly­(arylene sulfone sulfonate) (PASS), and polyphenylsulfone (PPSU)). (B) Distribution of graphene-based OSN membranes categorized by solvents used in performance evaluation (methanol (MeOH), ethanol (EtOH), isopropanol (IPA), DMF, acetonitrile (ACN), acetone (Ace), and toluene (Tol)). (C) Total number of graphene-based membranes reported for OSN and water-treatment applications. OSN performance comparison from Open and FAIR data sets for membranes tested in (D) EtOH and (E) IPA. Graphene-based membrane regions are highlighted with red circles.

**Table 1:** Summary of Physicochemical Properties, Membrane-Relevant Behaviors, and Major Industrial Uses of Representative Organic Solvents

solvent	ε (dielectric constant)	kinetic diameter (Å)	viscosity (cP)	boiling point (°C)	key functional behaviors (membrane & process)	major industrial applications
MeOH	32.6	3.8	0.544	64.6	• low viscosity → very high OSN permeance	fine chemicals & API synthesis, transesterification (biodiesel), HPLC/mobile phases
					• strong hydrogen bonding and high polarity → efficient sorption	
					• fast drying → ideal for cleaning	
Acetone	21.01	4.7	0.306	56.2	• very high diffusivity → high OSN permeance	semiconductor equipment cleaning, polymer dissolution, pharma reactions
					• moderate polarity → moderate swelling for many polar polymers	
					• fast drying → ideal for cleaning	
IPA	18.3	4.7	2.038	82.6	• moderate hydrogen bonding→ affects membrane sorption	wafer cleaning, drying, rinsing (UP/EL grade), battery electrode surface wash
					• excellent wetting of hydrophilic surfaces	
					• controlled drying	
Ethanol	24.6	4.4	1.074	78.5	• protic, strong membrane–surface interaction	extraction, recrystallization, bioprocessing
					• moderate swelling of polar polymers	
					• good solvation of organics	
Acetonitrile (ACN)	37.5	3.4	0.369	82	• high ε → penetrates polar polymers	HPLC, API synthesis, semiconductor rinse
					• very low viscosity → high OSN flux	
					• low residue → ideal for electronics	
Ethyl acetate (EA)	6	5.2	0.440	77	• intermediate polarity→ stable with hydrophobic membranes	extraction, pharma crystallization, OSN sweeps
					• good solvation of aromatics	
					• high permeance	
DMF	38.25	5.0	0.816	153	• strong swelling of amide/polyimide membranes	polymer synthesis, electronics stripping
					• slow transport in OSN due to large size/strong interaction	
					• solvates many polar polymers	
DMSO	47	6.5	1.99	189	• high ε → strong polymer swelling → decreased selectivity	advanced synthesis, semiconductor residue removal
					• high viscosity → low OSN flux	
THF	7.52	4.8	0.456	65	• swells hydrophobic polymers (PDMS, PI)	polymerization, Grignard reactions, battery binder processing
					• fast permeance in OSN	
					• strong organometallic compatibility	
PGMEA	8.3	4.6	0.8	146.4	• large molecular size → low permeance in OSN	photoresist solvent (semiconductor, OLED)
					• excellent film formation behavior	
NMP	32	5.1	1.89	202	• strong polymer swelling → lowers selectivity	Li-ion battery slurry, resist stripping, high-polar chemical reactions
					• high solvation for PVDF (battery binder)	
					• high boiling point → slow drying	
Toluene	4	5.9	0.555	110.6	• swell hydrophobic/aromatic polymers	petrochemicals, solvent for polymers and coatings
					• low ε → good stability for cross-linked, polar membranes	
					• good aromatic solvation	
Xylene	27	5.8	138.4	138.3	• high aromatic solvation power	BTX (refining), coatings, polymer processes
					• high boiling point → slow evaporation	
					• stable with cross-linked hydrophobic membranes	
Hexane	1.89	4.5	0.300	69	• very low ε → low swelling for polar membranes	oil extraction, rubber processing, OSN nonpolar separations
					• high flux due to low viscosity	
Cyclohexane	2.02	6.0	1.020	80.7	• good stability with hydrophobic membranes	Nylon precursor manufacturing, petrochemical reactions
					• medium viscosity → reduced flux	
DCM	8.93	4.9	0.414	39.8	• fast permeation	API purification, organic reactions
					• moderate polarity → controlled swelling	
					• very volatile	

**Table 2:** Comparison of Key Parameters Governing GO Membrane Operation in Organic Solvent and Aqueous Systems

parameters	organic solvent system	water solvent system
solvent properties	• wide polarity range (nonpolar–polar aprotic)	• high polarity and dielectric constant
	• low dielectric constant (except polar aprotics)	• strong hydrogen bonding
	• higher volatility and lower surface tension than water	• good interaction with GO layers
GO layer stability	• interlayer spacing highly sensitive to solvent polarity and solubility parameters	• hydrophilic swelling increases *d*-spacing
	• π–π interactions weakened in some solvents	• risk of delamination under high shear flow or insufficient adhesion to support
	• risk of delamination or collapse in aprotic solvents	
separation mechanism	• size exclusion dominant	• size exclusion + electrostatic (Donnan) effects
	• electrostatic effects less pronounced than in water	• more predictable pore hydration and charge interactions
permeance	•strongly solvent-dependent (viscosity, solubility parameter)	• good permeance driven by hydrated nanochannels
	•extremely fast permeance with nanopore generation and highly crystalline graphene	
chemical stability requirement	• must resist aprotic, aromatic, ketone, and ester solvents	• stability in neutral or mild pH water systems
	• requires cross-linking (amines, metal ions) or reduction	• generally less harsh than strong organic solvents
interlayer spacing control	• must suppress excessive swelling (covalent cross-linking, ionic bonding, intercalation, reduction degree control)	• hydration increases spacing in a controlled manner
fouling behavior	• organic fouling by dyes, pharmaceuticals, and oil molecules	• organic fouling + inorganic scaling
	• significant hydrophobic interactions	
	• highly crystalline graphene resists fouling	• hydrophilic GO layers resist some fouling
operation pressure	• 10–40 bar typical with graphene oxide	• 1–10 bar typical for NF
	• <5 bar with porous and crystalline graphene	
failure mechanisms	• layer delamination	• electrostatic adsorption of solute on the graphene surface
	• excessive swelling	• delamination of the selective layer
	• polymer support dissolution	• cake formation and scaling
	• compaction after long-term solvent permeation and pressure (flux decline)	
applications	• fine chemical purification	• water purification and desalination
	• pharmaceutical API recovery	• wastewater treatment
	• catalyst recycling (homogeneous/organometallic)	• removal of heavy metals, salts, PFAS
	• solvent exchange and solvent recycling	• natural organic matter (NOM) removal
	• dye separation in organic media	• food & biotechnology (protein separation)
	• polymer fractionation	• drinking-water production
	• organic reaction mixture cleanup	• metal recovery (Li, rare metal recycle)

This Review summarizes the material and structural characteristics of graphene-based OSN membranes and the underlying separation mechanisms. Then, we summarize recent studies on key structural engineering strategies and representative applications, followed by a discussion of practical challenges. Ultimately, a separation process perspective is provided to clarify the potential of graphene-based OSN membranes and to outline future research directions toward industrial implementation. Single-layer graphene is another type of graphene membrane, but we want to note that this review will focus on multilayer graphene-based OSN membranes, as the multilayer graphene membrane can be more feasible for practical applications due to the low cost of graphene and relatively easy manufacturing process.^44^

## 2 Synthesis and Fabrication of Graphene-Based Membrane

### 2.1 Graphene Oxide

GO-based membranes differ from other 2D material membranes (e.g., MXene and transition metal dichalcogenides) primarily in their intrinsic chemical functionality, tunable interlayer nanochannels, and scalable processability.^45–48^ Unlike 2D materials such as graphene or transition metal dichalcogenides (TMDs), which generally have fewer intrinsic surface functional groups, GO contains abundant oxygen-containing functional groups (epoxy, hydroxyl, and carboxyl), enabling hydrophilicity, strong intersheet interactions, and facile chemical modification.^49,50^ These features allow precise control of interlayer spacing, creating angstrom-scale nanochannels for selective molecular transport.^51^ GO membranes also exhibit solution processability, enabling large-area fabrication *via* vacuum filtration, casting, or slot die coating methods, which is advantageous for practical membrane engineering.^14,52^ Additionally, the presence of defects and functional groups facilitates selective transport mechanisms such as size sieving, electrostatic interactions, and adsorption–diffusion pathways.^53^ In contrast, many other 2D materials require complex synthesis processes and often lack intrinsic functional tunability.^50^ More importantly, while other 2D materials can be prepared in small amounts after a complicated purification process to remove unexfoliated mother particles, the graphene flakes (particularly GO) can be prepared in bulk scale without a process to remove unexfoliated and unoxidized particles.^54,55^

Graphene oxide (GO) is commonly synthesized *via* the modified Hummers’ method, in which strong oxidants partially oxidize graphite, introducing oxygen-containing groups such as epoxides and hydroxyls on the basal plane and carboxyl groups primarily at sheet edges.^55,56^ TEM and SAED analyses indicate that GO nanosheets are predominantly exfoliated to monolayers and retain discernible crystalline *sp* ^*2*^ domains alongside oxidized, hybridized regions, producing a chemically heterogeneous surface (Figure 3A).^57^ This partial oxidation preserves the graphitic lattice while providing excellent dispersibility in polar solvents, enabling solution-processed membrane fabrication (Figure 3B).^58^ Structurally, GO membranes form a two-dimensional and multilayer laminar structure composed of stacked nanosheets, and solvent transport proceeds primarily through interlayer capillaries. These nanochannels can act as molecular sieves, offering controllable separations *via* the effective interlayer spacing.

**Figure 3:**
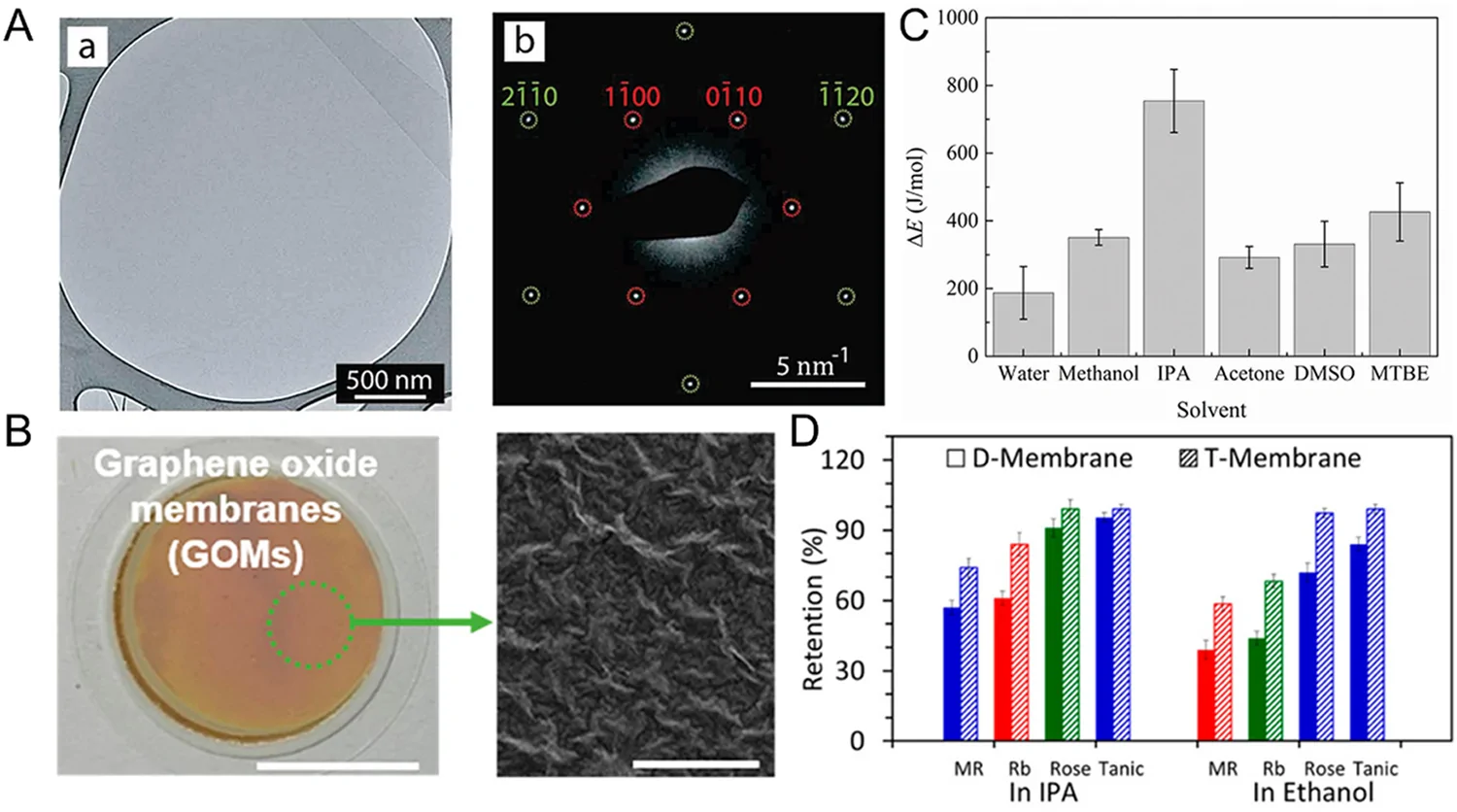
Structural characteristics and performance limitations of graphene oxide membranes. (A) Transmission electron microscopy (TEM) image of a single GO sheet and selected-area electron diffraction (SAED) of the GO representing a quasi-single crystal. Reproduced with permission from ref 57. Copyright 2009 American Chemical Society. (B) Photography and top-view SEM image of the GO membrane. Reprinted with permission from ref 58. Copyright 2021 American Chemical Society. (C) Free energy barrier to penetration of various solvents into the GO nanochannels. Reprinted with permission from ref 59. Copyright 2022 American Chemical Society. (D) Retention of various dyes in IPA and ethanol by the GO membrane before and after water soaking, highlighting the performance limitation of the GO membrane induced by disordered oxygen functional groups and uncontrolled swelling. Retention represents the percentage decrease in dye concentration from the feed to the permeate. Reprinted with permission from ref 17. Copyright 2018 American Chemical Society.

Because GO contains abundant oxygen-containing groups, solvent transport can be highly sensitive to membrane–solvent interactions. Molecular simulations on GO nanochannels suggest that permeation is governed through a combination of solvent molecular size, bulk viscosity, and solvent–membrane interactions (Figure 3C).^59^ Depending on solvent polarity, stronger solvent–membrane interactions in oxidized regions can induce local clustering and reduce molecular mobility. Moreover, unlike the atomically flat GO sheets often assumed in simulations, real GO exhibits corrugation and chemical heterogeneity, which can increase pathway tortuosity and interfacial friction, thereby raising effective transport resistance. By contrast, graphitic (*sp* ^*2*^) domains can provide comparatively low-friction pathways. Although GO laminates can exhibit high rejection in water, rejection may decrease in organic solvents because solvent-dependent swelling and weakened electrostatic/hydration-mediated gating reduce the effective sieving strength of the interlayer nanochannels. Therefore, the retention of the GO membrane after soaking in water (T-Membrane) showed higher retention compared with the GO membrane in the dried state (D-Membrane)^17^ (Figure 3D). Therefore, GO laminates should be engineered to simultaneously maintain effective sieving *via* swelling and defect control, and reduce transport resistance by limiting tortuosity and interfacial friction, thereby balancing rejection and permeance under OSN conditions.

### 2.2 Porous Graphene Oxide

In graphene-based laminates, solvent molecules must transport along interlayer channels, leading to long effective diffusion pathways and large mass-transfer resistance because of their barrier properties.^51^ To overcome this limitation, introducing nanopores on the graphene basal plane provides an additional solvent transport channel and can substantially enhance permeation.^60^ For single-layer graphene, nanopores have typically been generated *via* direct pore drilling methods such as plasma/ion bombardment or oxidative etching.^61–63^ In contrast, for multilayer GO/rGO laminates, pore generation has been achieved *via* various approaches, including thermal reduction, chemical treatments, photochemical, or electrochemical etching.^64–67^ For example, Hegab et al. produced holey graphene by oxidative chemical etching of GO using an H_2_O_2_/NH_4_OH mixture, thereby introducing abundant in-plane nanopores (Figure 4A).^68^ And Liu et al. reported a scalable nanowire-assisted electrochemical etching strategy, where nanowire-tip confinement effects induce localized oxidation and hole formation in GO nanosheets (Figure 4B).^69^ Among these, thermal reduction is one of the most widely used strategies. During thermal reduction, the desorption of oxygen-containing groups can release CO or CO_2_ and induce carbon-lattice rearrangement, generating vacancies and nanoscale pores.^64^ Pore-formation behavior and pore-size tendencies can be tuned by the initial oxidation degree and the functional-group distribution, such as the epoxy ratio (Figure 4C).^70^

**Figure 4:**
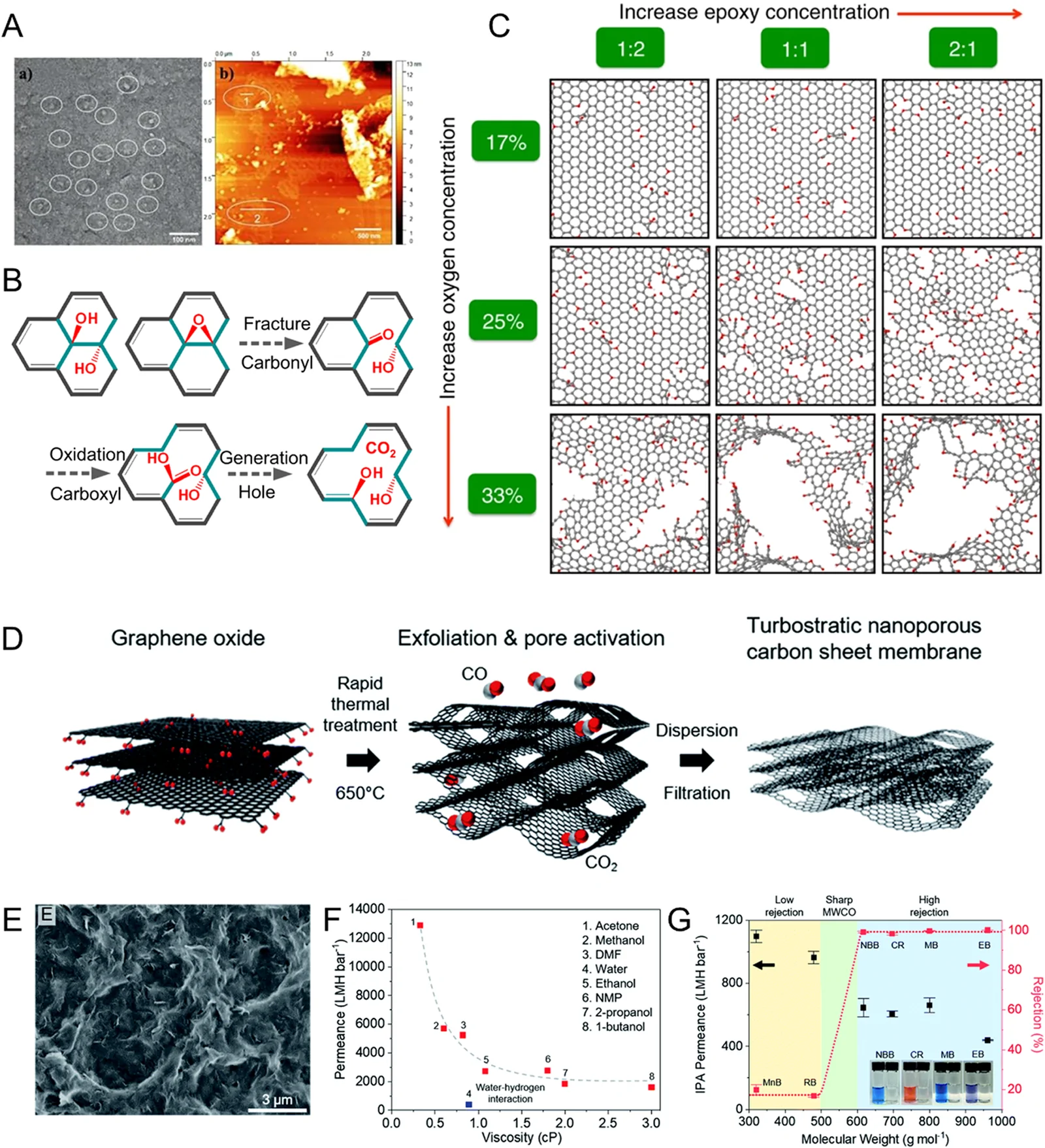
Synthesis, structure, and superior separation performance of porous reduced graphene oxide (rGO) membranes. (A) TEM and atomic force microscope (AFM) images of synthesized holey graphene nanosheets by chemical etching. Reprinted with permission from ref 68. Copyright 2022 Elsevier. **(B)** The role of oxygen functional groups (epoxy and hydroxy) during the graphene oxidation and reduction process in generating nanopores. Reprinted with permission under a Creative Commons CC BY 4.0 from ref 69. Copyright 2024 Springer Nature. (C) The influence of initial GO sheet properties, including oxygen concentration and epoxy/hydroxyl ratio, on the resulting rGO structure and nanopore size. Reprinted with permission under a Creative Commons CC BY 4.0 from ref 70. Copyright 2015 Springer Nature. (D) Schematic illustrating the fabrication method of the rGO membrane. (E) Scanning electron microscope (SEM) image of rGO membrane. (F) The high permeance of porous rGO membranes in a variety of solvents. (G) OSN performance of porous graphene membrane. Reprinted with permission from ref 40. Copyright 2020 The Royal Society of Chemistry.

Jang et al. demonstrated a porous GO-derived OSN membrane by applying rapid thermal treatment, producing a laminate of turbostratic nanoporous carbon sheets (TNCS) (Figure 4D).^40^ The pores created by the removal of oxygen groups, combined with the tendency of the layers to restore graphitic properties *via* stacking, result in a turbostratic structural morphology (Figure 4E). As a result, the TNCS membrane achieved ultrahigh permeance in pure IPA ∼1800 LMH/bar while maintaining a sharp MWCO around ∼600 Da, underscoring the value of coupling basal-plane porosity with stabilized stacked-sheet structures (Figure 4F,G). Introducing well-controlled nanopores transforms inherently tortuous, interlayer-limited transport into a dual-pathway transport, enabling high solvent throughput while preserving sharp molecular sieving in OSN.

### 2.3 Crystalline Porous Graphene Oxide

Pore generation, especially thermal pore activation, is one of the powerful approaches to enhance solvent permeability. However, this leads to an amorphous carbon structure, which can deteriorate the transport of solvent molecules.^22,42^ Kang et al. introduced microwave-assisted crystallization, which restores local graphitic order, including the recovery of *sp* ^2^ carbon domains and hexagonal in-plane ordering in multilayer nanoporous graphene (Figure 5A).^22^ Nanopores are first created by low-temperature thermal activation of GO, yet the activated nanoporous graphene (NG) still contains disordered amorphous *sp* ^*3*^ regions due to insufficient recrystallization.^13,42^ This structural disorder can introduce additional friction and heterogeneity in nanochannels, limiting the intrinsic advantage of graphene nanoconfinement for fast solvent transport. Microwave irradiation addresses this trade-off by inducing an extremely high local temperature on the conductive carbon framework, enabling recovery of crystalline *sp* ^2^ domains while maintaining (and even enhancing) nanopore density (Figure 5A). Raman spectroscopy shows a sharpened *D* band along with the emergence of the *2D* peak, indicative of long-range ordered *sp* ^2^ carbon, while the *D*/*G* ratio increases because nanopore formation introduces a high density of edge sites (Figure 5B). Consistently, HR-TEM directly visualizes the restoration of a hexagonal graphene lattice after microwave treatment, confirming the transformation from *sp* ^3^ to *sp* ^2^ (Figure 5C).

**Figure 5:**
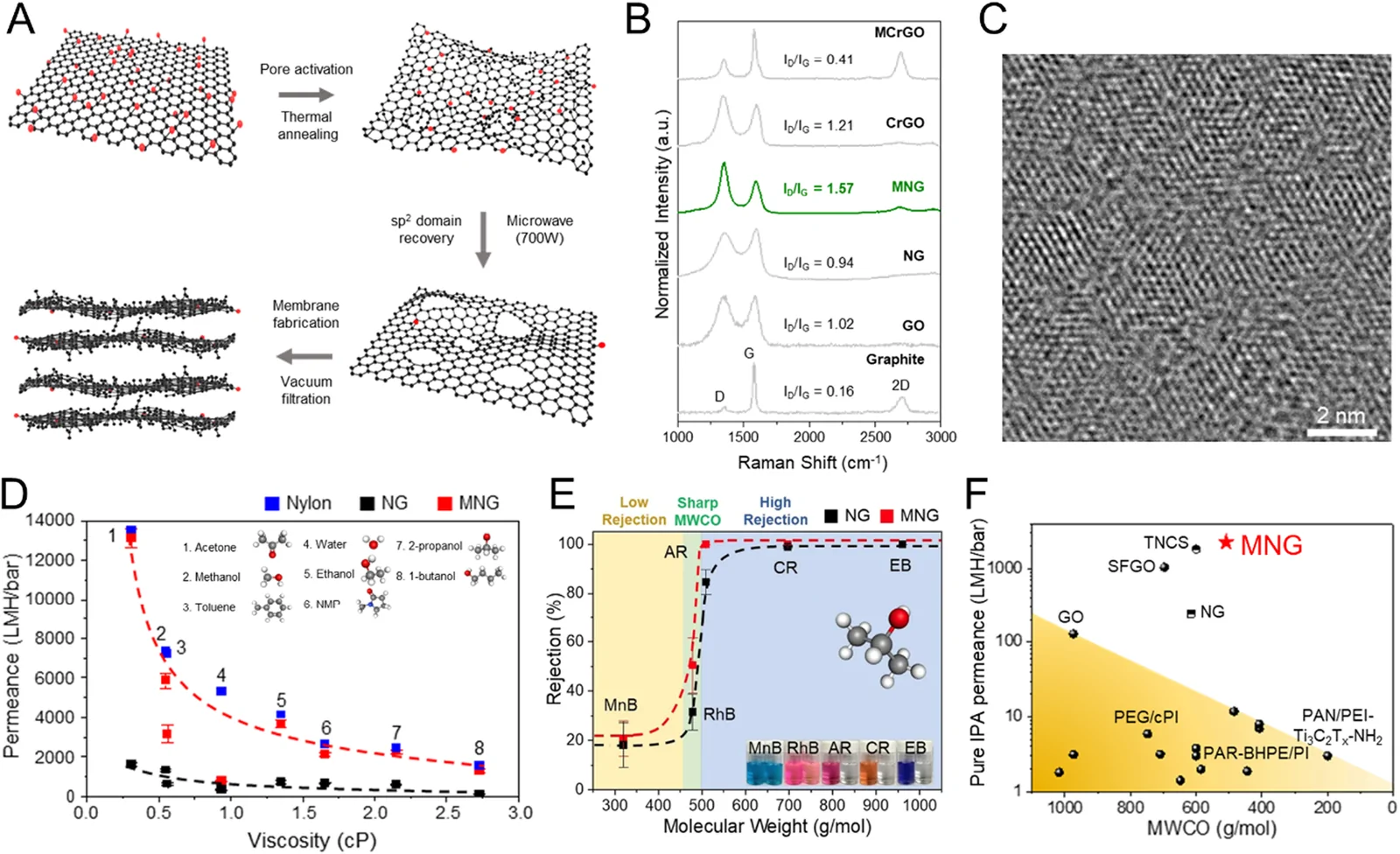
Crystalline multilayer nanoporous graphene. (A) Schematic illustration of the fabrication of microwave-treated nanoporous graphene, showing pore activation by thermal annealing of GO, subsequent microwave-assisted recovery of *sp* ^*2*^ carbon domains with additional pore generation. (B, C) Characteristic properties of *sp* ^*2*^-rich multilayer nanoporous graphene: Raman spectra (B) and high-resolution transmission electron microscopy (HR-TEM) image (C). **(D–F)** OSN performance of crystalline multilayer nanoporous graphene: Ultrafast organic solvent permeance (D), precise molecular sieving (E), and high OSN performance compared to other polymeric and 2D material membranes. Reprinted with permission under a Creative Commons CC BY 4.0 from ref 22. Copyright 2023 Springer Nature.

At the membrane level, the recovery of *sp* ^*2*^ crystallinity promotes a more compact and well-aligned lamellar architecture, resulting in narrower and more ordered interlayer nanochannels than those in noncrystalline porous graphene. This structural ordering preserves the nanoporous framework while substantially improving transport efficiency and overall membrane performance, as the *sp* ^*2*^-rich, atomically smooth nanochannels provide low-friction pathways for solvent transport. The resulting microwave-treated NG (MNG) membrane exhibits exceptionally high solvent permeance, ranging from 13,117 LMH/bar for acetone to 1300 LMH/bar for 1-butanol, approaching the permeance of the bare nylon support (Figure 5D). At the same time, compared with nonmicrowave-treated NG membranes, the MNG shows enhanced rejection in the sub-600 Da range, which can be attributed to the formation of a more compact and well-aligned lamellar structure enabled by crystallinity recovery (Figure 5E). As a result, the MNG membrane clearly surpasses the conventional permeance–selectivity upper bound for OSN, simultaneously achieving ultrahigh permeance and low MWCO (Figure 5F). These results highlight that preserving nanopores while restoring graphene crystallinity is a key strategy to overcome the typical trade-off between permeability and selectivity in OSN membranes.

## 3 OSN Mechanism for Graphene-Based Membrane

### 3.1 Role of Nanopore and Interlayer Spacing for Molecular Separation

In multilayer nanoporous graphene membranes, solvent permeation proceeds *via* two concurrent pathways: direct transport through nanopores and confined transport along interlayer nanochannels.^71,72^ Figure 6 summarizes simulation-based studies that clarify how these transport modes collectively determine permeation behavior, as revealed by molecular dynamics (MD) simulations.^22,71^ In the pore-mediated pathway (Figure 6A), simulations demonstrate that nanopores serve as efficient through-plane shortcuts that significantly enhance solvent permeance by reducing the effective transport length across the stacked graphene layers. As pore size increases, solvent permeability increases accordingly (Figure 6B); however, this study consistently indicates the existence of an optimal pore-size window (greater than 2 nm) in which permeance is maximized without compromising molecular selectivity. Excessively large pores can form nonselective transport pathways when aligned across multiple layers, underscoring the importance of precise pore-size control.

**Figure 6:**
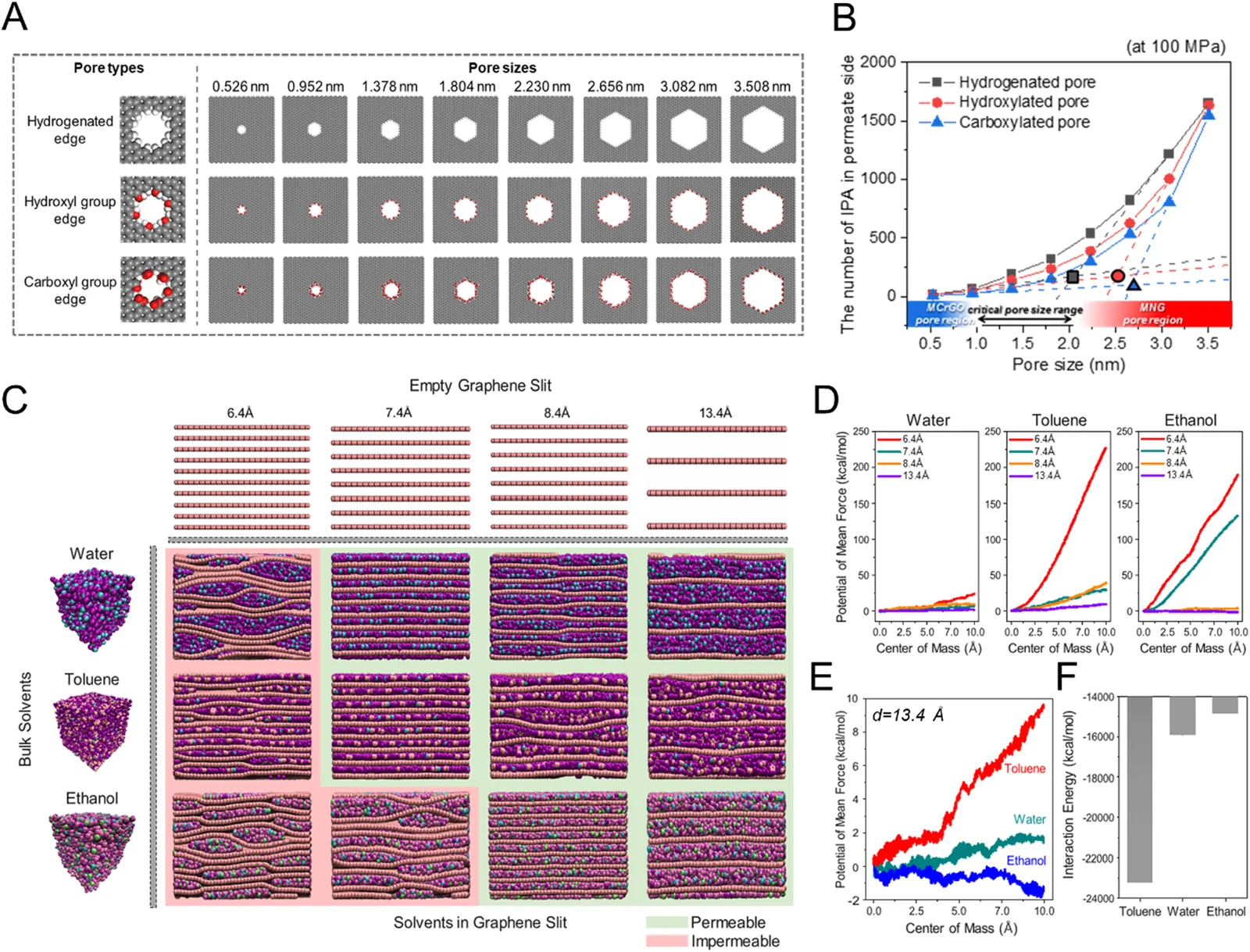
Molecular transport on multilayer nanoporous graphene. (A) Graphene nanopore models with different edge chemistries. (B) Solvent permeation according to pore size and functional groups. Reprinted with permission from ref 71. Copyright 2025 Elsevier. (C) Solvent permeation through graphene layers with different interlayer spacings. (D) Potentials of mean force of solvents. (E) Potentials of mean force of water, toluene, ethanol, and (F) interaction energy associated with graphene–solvent interactions at the *d*-spacing of 13.4 Å. Reprinted with permission under a Creative Commons CC BY 4.0 from ref 22. Copyright 2023 Springer Nature.

In parallel, the interlayer-mediated pathway (Figure 6C) captures solvent transport within graphene interlayers, where confinement effects dominate transport resistance. Steered MD simulations quantify this behavior through the potential of mean force (PMF), revealing that solvent permeability is highly sensitive to interlayer spacing. Narrow spacings impose substantial free-energy barriers due to limited accessible volume, whereas increased spacings lower these barriers and facilitate smoother solvent migration (Figure 6D,E). Furthermore, solvent-dependent PMF profiles correlate strongly with solvent–graphene interaction energies (Figure 6E,F), indicating that stronger interactions lead to higher transport resistance within the nanochannels. Together, these simulation studies highlight that efficient molecular transport in multilayer nanoporous graphene membranes arises from the synergistic interplay between nanopore-mediated through-plane transport and low-resistance interlayer diffusion, providing a mechanistic framework for understanding and optimizing OSN membrane performance.

### 3.2 Solvent–Membrane Interactions

Solvent–graphene-based material affinity significantly influences OSN performance by conditioning both the effective interlayer nanochannel and the interfacial transport resistance.^73,74^ Zheng et al. systematically correlated GO swelling in organic solvents with solubility parameters (Figure 7A).^75^ Good solvents that interact favorably with GO tend to induce pronounced swelling and expanded interlayer spacing, whereas solvents that are less compatible with GO maintain relatively narrow, size-selective nanochannels (Figure 7B). In particular, the Hansen solubility distance (SD) serves as a practical predictor. Solvents with SD < 9.5 generally cause substantial swelling (interlayer spacing up to ∼2.7 nm), while solvents with SD > 9.5 exhibit only minor swelling and maintain an interlayer spacing of ∼1 nm, which can preserve high dye rejection in selected solvents.

**Figure 7:**
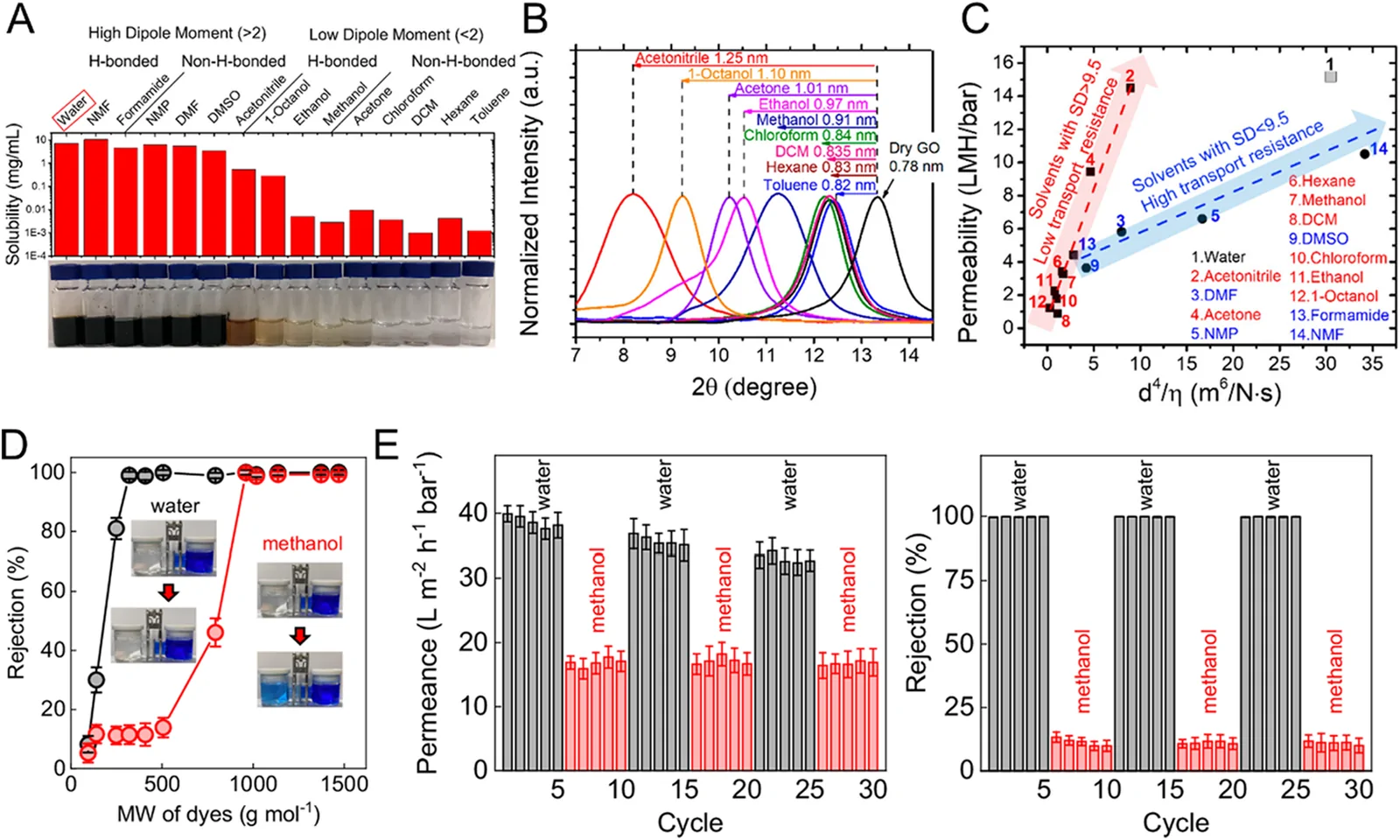
(A) Solubility of GO in various solvents. (B) Interlayer spacing of GO membranes after immersion in various solvents. (C) Correlation between solvent permeance of GO membrane and two key solvent descriptors-d^4^/η (transport parameter) and Hansen solubility distance (SD). Reprinted with permission from ref 75. Copyright 2020 American Chemical Society. (D) MWCO curves of GO/porous graphene (PG) membranes in water and methanol. (E) Reversible changes in solvent permeance (left) and dye rejection (right) during repeated switching between water and methanol feeds. Reprinted with permission under a Creative Commons CC BY-NC-ND 4.0 from ref 78. Copyright 2025 Springer Nature.

When permeance is plotted against the integrated hydrodynamic parameter d^4^/η, two distinct regimes appear, not following a simple viscosity-based trend (Figure 7C). Solvents with large SD (>9.5) show a steeper permeability–d^4^/η dependence, consistent with a lower effective transport resistance. By contrast, solvents with small SD (<9.5) exhibit a much gentler slope, indicating a higher effective transport resistance. This deviation is attributed to solvent-dependent boundary slip in GO nanochannels, where stronger solvent–GO interactions increase interfacial friction and reduce slip, whereas higher-SD solvents exhibit high slip velocities, enhancing permeance despite narrower channel spacing.

The correlations discussed above were primarily established for relatively homogeneous GO laminates. However, solvent affinity and swelling can vary substantially with the GO state, including the degree of functional groups, defect density, and crystallinity, which collectively define the effective nanochannel chemistry and boundary conditions.^16,76,77^ Chemically heterogeneous graphene-based membranes further introduce complexity and responsiveness. Li et al. demonstrated that incorporating PG into GO laminates creates multiple transport environments within a single membrane.^78^ The heterogeneous system employs solvent-specific interactions to drive solvent-dependent pathway selection. In Figure 7D, switching the solvent from water to methanol triggers a reversible shift in the MWCO from 319 to 960 Da. This occurs because water prefers the tighter GO–GO channels, whereas methanol, due to specific affinity and confinement effects, drastically expands the GO–PG channels, making them the dominant transport pathway. This enables reversible switching in both permeance and rejection upon solvent cycling (Figure 7E), demonstrating that solvent–membrane interactions in heterogeneous channel networks can reversibly shift the dominant transport pathway and OSN performance.

## 4 Structural Engineering Strategies

### 4.1 Pore Sealing

In porous single-layer graphene, various pore-sealing and pore-refinement strategies, such as chemical functionalization, polymer deposition, metal/oxide deposition, and molecular adsorption, have been widely employed to suppress nonselective leakage through oversized pores while retaining high permeance.^79–82^ These approaches, originally developed for atomically thin membranes, provide a conceptual foundation for pore regulation and can be extended to the multilayer nanoporous graphene structure, where uncontrolled pores pose an even greater challenge due to cumulative through-plane alignment.

In multilayer nanoporous graphene membranes, precise pore structure control is a critical requirement because oversized or nonuniform nanopores can create nonselective transport pathways that undermine molecular sieving, even when well-defined interlayer nanochannels are present.^71^ While nanopores are essential for achieving a high permeance, their dimensions and distribution must be carefully regulated to maintain selectivity in practical OSN applications.

To address this challenge, Kang et al. introduced an organometallic precursor-assisted atomic layer deposition (ALD) strategy to refine the pore structure of multilayer nanoporous graphene membranes (Figure 8A).^83^ By selectively depositing alumina at pore edges and defect sites, this approach enables controlled narrowing and sealing of nonselective surface nanopores while preserving the underlying nanoporous graphene framework (Figure 8B). At the same time, partial infiltration of the precursor into the upper graphene layers induces alumina-mediated interlayer cross-linking, which enhances the mechanical and solvent-induced structural stability of the lamellar architecture. As a result, membrane selectivity can be systematically enhanced without eliminating the high-flux transport pathways provided by the nanopores.

**Figure 8:**
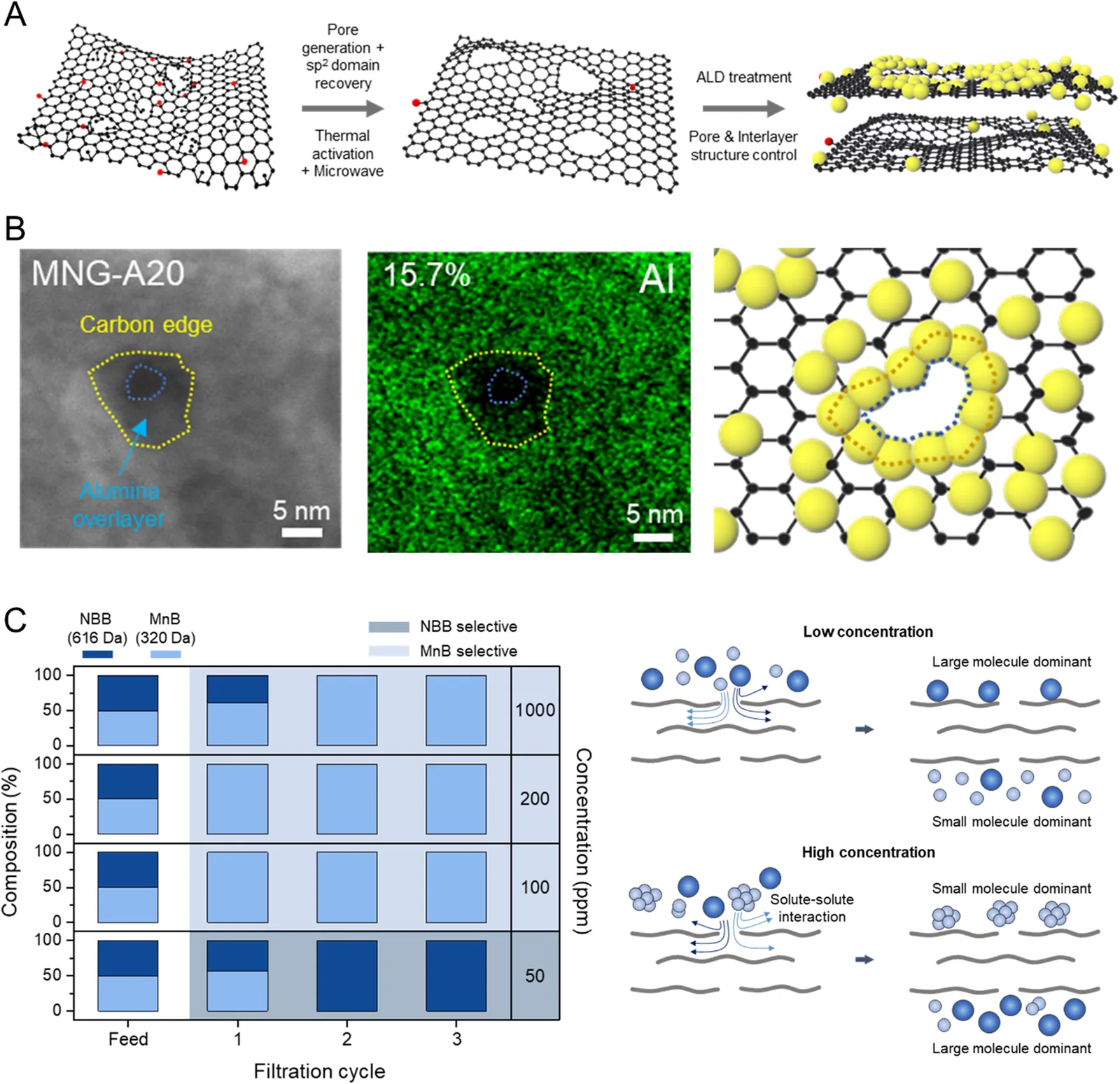
Pore structure control of multilayer nanoporous graphene. (A) Organometallic precursor-induced pore structure control approach. (B) Narrowed graphene surface nanopore: High-angle annular dark-field scanning transmission electron microscopy (HAADF-STEM) image (left), corresponding energy-dispersive X-ray spectroscopy (EDS) mapping image (middle), and a schematic of pore tuning (right). (C) Binary mixture separation with molecules smaller than the membrane’s MWCO. Adapted with permission from ref 83. Copyright 2025 The Authors. Published by Wiley-VCH GmbH under a Creative Commons Attribution Non-Commercial No Derivatives License (CC BY-NC-ND).

Importantly, this pore-engineering strategy not only improves conventional MWCO-based selectivity but also enables effective separation of binary mixtures even when both solutes are smaller than the nominal MWCO (Figure 8C). The effective solute size can be changed according to concentration due to solute–solute interactions and transient confinement. As a result, repeated membrane filtration can progressively enhance rejection and product purity. This behavior demonstrates that structural pore refinement and interlayer stabilization can translate into reliable performance under realistic multicomponent conditions. Overall, Figure 8 illustrates that pore engineering in multilayer nanoporous graphene membranes is not only a tool for boosting performance but also a key strategy for enabling precise and practically relevant molecular separations, providing important design guidance for next-generation OSN membranes.

### 4.2 Interlayer Spacing Regulation

In laminar graphene-based membranes, the interlayer spacing refers to the distance between the adjacent graphene sheets. It not only governs the selectivity of the membrane but also strongly influences permeance through frictional and viscous resistance within the confined channels.^11,53,84^ In particular, GO laminates can maintain relatively compact stacking in the dry state; however, under OSN conditions, solvent uptake and swelling, facilitated by abundant oxygen functionalities, can readily alter the *d*-spacing.^75^ Therefore, the channel width can be dynamically modulated depending on solvent identity (polarity and hydrogen bonding), concentration, and operating conditions, leading to changes in the performance.^17,76,85,86^ For these reasons, interlayer spacing is regarded not only as a structural feature but also as a key design variable that determines the separation performance and long-term stability in graphene-based membranes, motivating diverse strategies to precisely tune *d*-spacing.

Among these strategies, intercalation-based regulation has become one of the most widely adopted design approaches for laminar graphene-based membranes (Figure 9A–C). Intercalators are introduced into the graphene interlayers to modulate the *d*-spacing and lamellar packing, thereby altering the effective transport pathway and hydraulic resistance. Accordingly, various types of intercalators have been explored, including 0D nanoparticles/MOF crystallites,^68,87–90^ 1D fibrous/nanotubular/nanowire networks,^91–95^ and 2D nanosheets (e.g., MXene and MoS_2_).^96–98^ For 0D intercalators, Chen et al. achieved in situ intercalation of ZIF-8 nanocrystals within GO interlayers to form enlarged nanochannels, reporting acetone permeance of ∼10,030 LMH/bar and methanol permeance of ∼6816 LMH/bar while maintaining high rejection for organic molecules (MW > 900 Da) (Figure 9A).^87^ Similarly, Wang et al. further demonstrated that the channel size in ZIF-8–GO membranes could be expanded using spacing expanders (quaternary ammonium salts), enabling higher flux for *n*-hexane-based hydrocarbon mixture separation and highlighting applicability in nonpolar systems.^88^ For 1D intercalation, the incorporation of nanotubes/nanowire networks between GO sheets primarily serves to expand the interlayer spacing, while simultaneously altering the packing and connectivity of the lamellar channels. In unzipped-CNT embedded GO (uCNTm), the laminate architecture was preserved after incorporation of an uCNT network, while the *d*-spacing showed only a modest change from 0.76 to 0.78 nm (Figure 9B).^92^ Extending to OSN, Nie et al. reported that incorporating CNTs into GO-based laminar channels increased ethanol permeance to ∼138 LMH/bar (with high dye rejection) and maintained permeance of ≥60 LMH/bar in butanol.^93^ In 2D intercalation, heterostacking is formed by inserting secondary 2D building blocks into the GO interlayers, thereby reconstructing the channel geometry. Geng et al. assembled small MoS_2_ nanosheets with GO as spacers and, at an identical mass density, achieved acetone permeance of 436 LMH/bar and water permeance of 744 LMH/bar (Figure 9C).^96^ Compared with the pristine GO membrane, the acetone and water permeance increased by approximately 55-fold and 27-fold, respectively, while maintaining high rejection. Overall, intercalation is most often employed to enlarge the interlayer spacing of laminar graphene membranes, thereby increasing solvent permeance.

**Figure 9:**
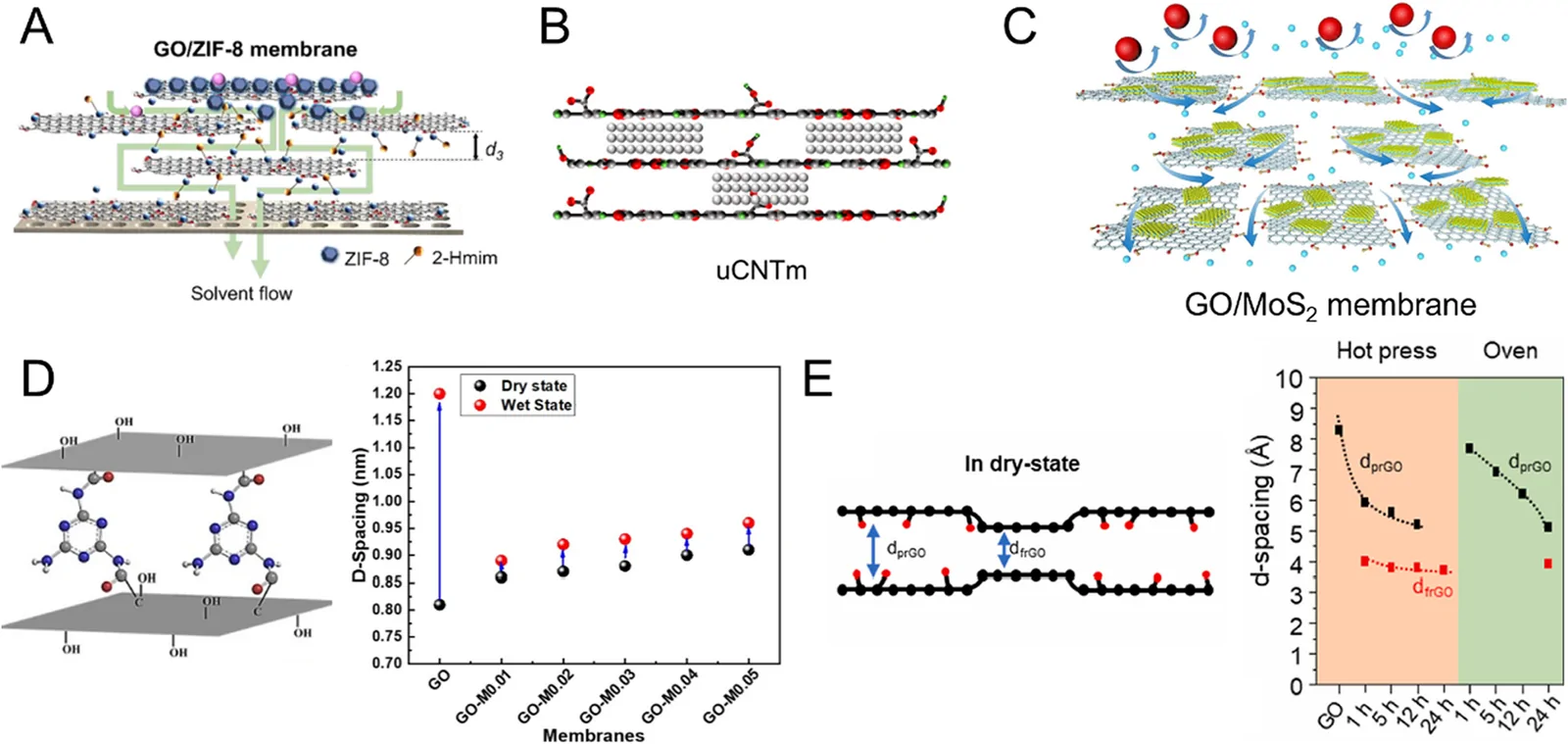
Interlayer Spacing Regulation. (A) Schematic illustration of GO/zeolitic imidazolate framework-8 (ZIF-8) membrane. ZIF-8 nanocrystals and 2-methylimidazole (2-Hmim) were intercalated in the GO interlayer. Reprinted with permission from ref 87. Copyright 2021 Elsevier. (B) Schematic illustration of an unzipped-CNT embedded GO membrane (uCNTm). Reprinted with permission from ref 92. Copyright 2022 American Chemical Society. (C) Schematic illustration of GO/Molybdenum disulfide (MoS_2_) membrane. Reprinted with permission from ref 96. Copyright 2023 Elsevier. (D) Schematic illustration of melamine cross-linked GO membrane. Reprinted with permission from ref 100. Copyright 2024 Elsevier. (E) Schematic illustration nanoporous multilayer GO membrane and post-treatment for interlayer regulation. Reprinted with permission from ref 21. Copyright 2023 Elsevier.

However, enlarged interlayer spacing can be accompanied by increased solvent-induced swelling under OSN conditions. In other words, increased *d*-spacing in the dry state is not necessarily preserved during OSN operation because solvent-induced swelling and microstructural reorganization can alter the effective channel width and, thus, selectivity.^16,17,75^ Therefore, some researches have focused not only on increasing *d*-spacing but also on maintaining that spacing under operating conditions. Cross-linking is widely used to immobilize GO interlayers, thereby regulating *d*-spacing and mitigating solvent-induced swelling in OSN.^99–102^ Nakagawa et al. introduced triethanolamine (TEOA) as a cross-linker that forms hydrogen-bond-mediated bridges between GO sheets, thereby stabilizing the GO laminate and suppressing swelling.^99^ In Figure 9D, Abebe et al. further demonstrated that melamine incorporation (GO–M0.02) markedly improves interlayer stability in the wet state. Melamine cross-links GO through the reaction of its amine groups with the carboxyl and epoxide groups on the GO surface, thereby forming covalent bonds that stabilize the lamellar structure.^100^ Consistently, the ethanol flux remained nearly constant at ∼ 22 LMH over 48 h while maintaining methyl green rejection above 98%, supporting improved operational stability. In addition, post-treatments such as thermal treatment and hot pressing have also been explored to physically reorganize the lamellar structure and moderate *d*-spacing.^21,39^ Kim et al. employed hot pressing to partially reduce GO, lowering transport resistance while promoting alignment and densification of the laminate (Figure 9E).^21^ Compared with oven treatment at similar processing times, hot pressing provides stronger driving forces that enable faster stabilization of the interlayer structure. This structural regulation increased ethanol permeance of ∼ 300 LMH/bar while preserving a sharp MWCO of ∼ 500 Da. Furthermore, this work demonstrated stable long-term operation for 30 days under high ethanol permeance conditions due to the welding effect, underscoring that post-treatment can simultaneously support resistance reduction, structural stabilization, and practical OSN applicability.

### 4.3 Orientation and Aspect Ratio Control

In graphene-based membranes, OSN performance is governed not only by the interlayer spacing but also by nanosheet alignment, which determines channel continuity and tortuosity.^14,17,103^ Although an ideal structure can be described as a stack of uniform nanochannels, real membranes exhibit heterogeneous microstructural features such as interflake boundaries, wrinkles, and edge regions. The spatial arrangement of these features alters the effective transport path length and the density of nonselective pathways.^12,15^ As a result, changes in alignment can shift the balance between permeance and rejection even at the same material composition and thickness. Moreover, under OSN conditions, solvent–membrane interactions can alter nanosheet alignment, thereby changing the effective channel distribution and ultimately the separation performance.^16,17,104^ These considerations have motivated various approaches that engineer nanosheet alignment to control transport and selectivity.

Nanosheet orientation of the graphene-based membrane refers to the angular distribution of nanosheets relative to the support surface, which dictates channel connectivity and the density of nonselective pathways. This is particularly relevant in membrane fabrication, where the support surface morphology and GO–support interfacial affinity govern stacking behavior, allowing identical GO to yield markedly different channel structures depending on the substrate (Figure 10A). Gan et al. showed that the stacking alignment of GO varies systematically with substrate characteristics.^105^ Larger pore sizes and higher surface roughness, along with weaker GO–substrate interactions, promoted more disordered deposition, leading to larger nanochannel structures. Such substrate-driven differences in GO orientation can translate directly into OSN performance. Guan et al. reported that variations in surface pore size and porosity of the support altered GO stacking behavior and resulted in distinct methanol permeance and rejection (Figure 10B).^106^ For a nylon (NY) substrate with larger pores and higher porosity, pronounced wrinkles and nonselective defect pathways were formed, yielding higher methanol permeance but lower dye rejection compared with polyethersulfone (PES) and polyketone (PK) supports. Notably, these performance differences were observed without a substantial change in *d*-spacing, underscoring that wrinkle formation and stacking uniformity can be important factors under OSN conditions. Beyond substrate effects, several studies have further shown that GO–solvent affinity and fabrication methods can also alter microstructure and transport characteristics.^107,108^ Overall, these studies show that nanosheet orientation can be tuned and used to tailor transport and selectivity in graphene-based membranes under OSN conditions.

**Figure 10:**
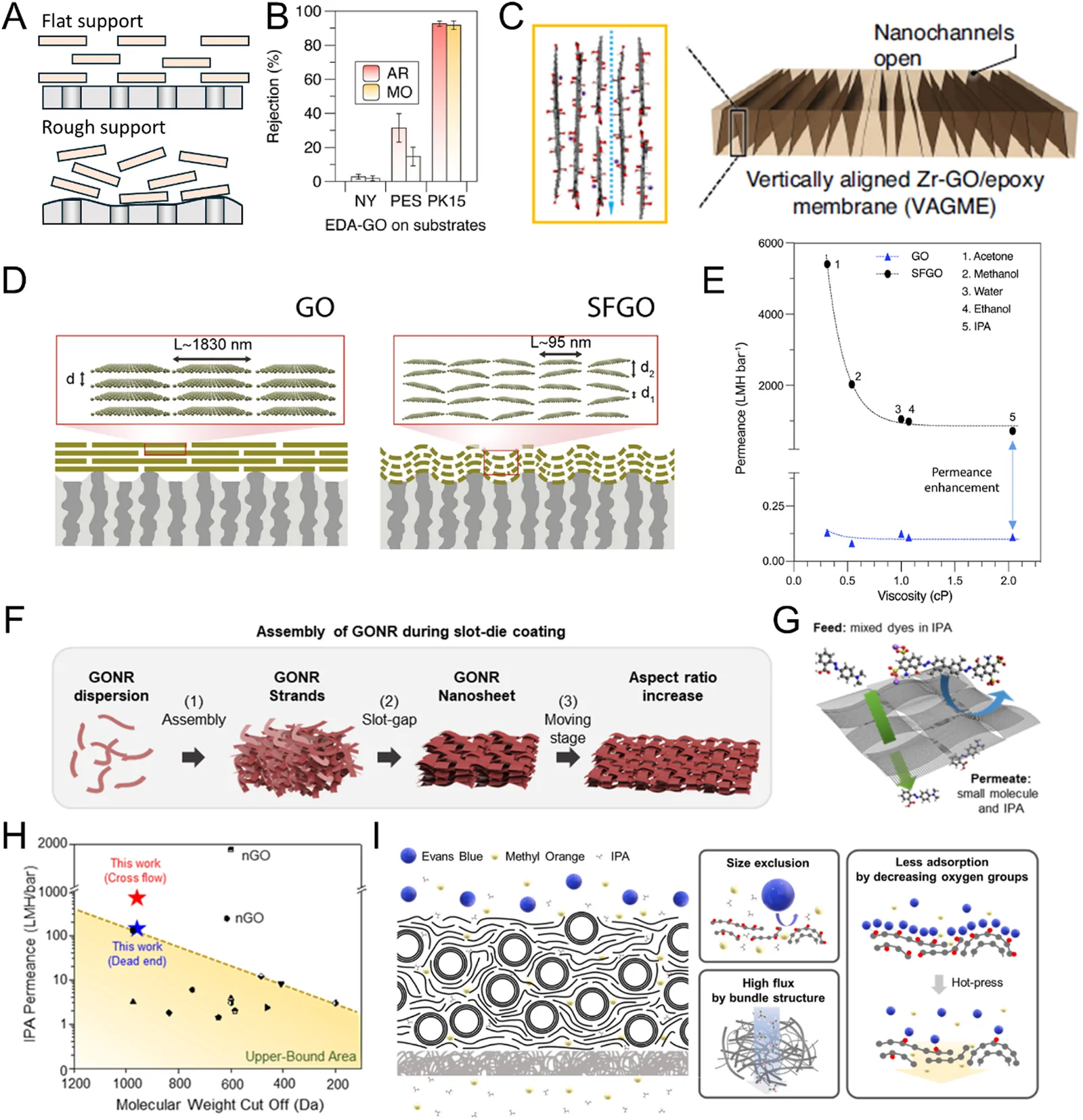
Orientation and Aspect Ratio Control. (A) Schematic illustration of the effect of substrate surface morphology on the stacking alignment of GO nanosheets. (B) Dye rejection performances of the GO-based membrane depending on the substrates. Reprinted with permission from ref 106. Copyright 2022 Elsevier. (C) Schematic illustration of a quasi-vertically asymmetric channel in the GO membrane. Reprinted with permission under a Creative Commons CC BY 4.0 from ref 109. Copyright 2021 Springer Nature. (D) Comparison of large-flake and small-flake GO (SFGO) membranes, and (E) permeance of GO and SFGO membranes in various organic solvents. Reprinted with permission from ref 41. Copyright 2021 Elsevier. (F) Schematic image of the GONR membrane fabrication process by sheet-shaping assembly. Entangled GONR strings are transformed into nanosheets under shear and confinement in the slot-die microgap. (G) Schematic illustrating the structure of a high-aspect-ratio GNR membrane. (H) Comparison of OSN performance in IPA with GONR membrane performance highlighted by stars. Reprinted with permission from ref 43. Copyright 2022 Elsevier. (I) Schematic illustration of CNT/GONR hybrid membrane, showing CNT intercalation between stacked GONR layers and their molecular separation mechanism. Reprinted with permission from ref 39. Copyright 2024 Elsevier.

Alongside the conventional lamellar membrane, some studies have proposed shortening the transport path length by orienting channels closer to the perpendicular direction. In 2021, Liu et al. introduced the concept of vertical nanochannels to reduce the effective pathway along the *Z*-direction (Figure 10C).^109^ Conventional GO laminates align nanosheets parallel to the substrate, resulting in long tortuous pathways. To address this, their approach reoriented nanosheet domains to form *Z*-directional transmembrane nanochannels. In the vertically aligned graphene membrane (VAGME), wrinkled or zigzag-shaped GO domains were immobilized in an epoxy matrix and sectioned into thin membranes, enabling near-vertical segments that shortened the pathway while retaining size-selective transport. However, the epoxy matrix can introduce additional flow resistance, so the permeance remained moderate despite the reduced geometric path length. Later, Han et al. reported quasi-vertically asymmetric channels by inserting and stacking GO flakes within a mixed cellulose ester (MCE) support with asymmetric pores, combining shorter, less tortuous pathways with a high channel density.^110^ These approaches illustrate that orientation control can extend beyond improving in-plane alignment within a lamellar stack to reconfiguring the transmembrane transport structure.

As another approach, graphene-based membranes have explored control of the building-block aspect ratio to tune the continuity of the laminate and the effective transport pathway (Figure 10D–I). In particular, regulating the lateral size distribution of 2D GO flakes has been widely investigated because it changes the frequency of interflake junctions and the overlap length between adjacent flakes, thereby modulating pathway tortuosity.^12,41,111,112^ Nie et al. used small-flake GO under organic-solvent conditions, suggesting that lateral-dimension control can promote shorter and less tortuous transport pathways.^12^ Furthermore, Ruiz-Torres et al. reduced the GO flake size *via* sonication to obtain SFGO and formed a thin selective layer that closely conformed to a rough nylon support (Figure 10D).^41^ This design increased the accessible area and substantially reduced the transport length and tortuosity. The SFGO membrane exhibited ultrafast permeance of 720–5410 LMH/bar across multiple solvents (acetone, methanol, water, ethanol, and IPA), far exceeding conventional GO membranes that typically show permeance on the order of 0.08–0.13 LMH/bar under similar loading conditions (Figure 10E). Overall, these studies highlight small-flake engineering as a simple way to tune interflake connectivity and pathway tortuosity, enabling highly permeable GO laminates.

In addition to tuning 2D flake size, membranes based on 1D building blocks have been developed to form interconnected transport networks through entangled assemblies. Graphene nanoribbons (GNRs) are 1D carbon materials that exhibit the structural features of graphene and carbon nanotubes (CNTs).^113^ GNRs contain strips of graphene with widths typically ranging from a few nanometers to less than 100 nm. This geometry creates a high-aspect-ratio nanostructure that differs fundamentally from the characteristics of 2D graphene sheets. As a result, GNRs can be stacked and form a narrow interlayer spacing owing to their graphene-like basal plane, and they can easily form entangled structures because of their high aspect ratio and narrow widths. Kim et al. demonstrated that laminated GNR membranes could deliver high solvent flux with limited sensitivity to solvent polarity (toluene: ∼975 LMH/bar, hexane: ∼240 LMH/bar).^114^ They also maintained high dye rejection and mechanical stability under high pressure (up to 50 bar), establishing nanoribbon laminates as a viable building block for pressure-driven separations. Subsequently, Choi et al. reported that graphene oxide nanoribbon (GONR) hydrogels exhibit shear-thinning behavior suitable for large-area coating, and that the entangled network formed during coating can preserve its morphology under high pressure and cross-flow operation, emphasizing the processability and structural robustness enabled by 1D building blocks.^115^ This concept was further extended to slot die fabrication, where nanoribbon assemblies were tuned to form a selective layer (Figure 10F).^43^ During slot die coating, spontaneous self-assembly occurred while the GONR dispersion passed through the confined microgap of the slot die head. The resulting GONR layer, unlike conventional GO layers, formed an entangled network architecture (Figure 10G), thereby increasing the connectivity of transport pathways across stacked domains. The GONR membrane achieved a high IPA permeance of 679 LMH/bar with an MWCO of 961 Da, indicating that 1D-based laminates can offer a shorter transport pathway (Figure 10H).^43^ Further, Kim et al. incorporated CNTs into CNT/GONR hybrid membranes, introducing CNTs as 1D intercalators within the nanoribbon layer to promote continuous transport pathways. With additional hot pressing to reduce the contribution of oxygen functionalities, the membrane exhibited alcohol permeance in the range of ∼60–200 LMH/bar while maintaining an MWCO of ∼961 Da (Figure 10I).^39^ It further demonstrated extended cross-flow stability (1315 h), attributed to enhanced adhesion between the selective layer and the support through a polymer welding effect. Collectively, these studies suggest that nanoribbon-based membranes can deliver ultrafast transport while also supporting practical requirements such as scalable fabrication, operation under high pressure, and long-term cross-flow stability in organic-solvent environments.

## 5 Functional and Industrial Applications

A variety of unit operations have been employed for purification, concentration, and solvent exchange/recovery, including distillation, solvent extraction, adsorption, chromatography, evaporation, and crystallization, and the optimal operation depends on the process objectives. On the basis of comparison of these representative separation technologies (Table 3), we discuss how graphene-based OSN membranes can be applied for purification, concentration, and solvent exchange/recovery.

**Table 3:** Summary of Separation Principles, Energy Requirements, Mechanisms, and Applications of OSN and Other Major Solvent-Processing Techniques

method	driving force	energy demand	mechanism	phase change	characteristics	application
OSN	pressure difference (Δ*P*)	low–medium (pump work)	solution–diffusion, size/interaction-based rejection	no	moderate selectivity, high throughput, sensitive to solvent stability, and swelling	API concentration, solvent exchange/polishing, polymer/oligomer removal
solvent Extraction	distribution coefficient, chemical potential difference between phases	low–medium (mixing, phase disengagement)	equilibrium partitioning between immiscible liquid phases	no	high selectivity via solvent choice; simple operation; requires phase separation	product extraction, impurity removal, metal/organics extraction
distillation	volatility difference, temperature gradient, vapor–liquid equilibrium	high–very high (latent heat for vaporization/condensation)	repeated vaporization–condensation, relative volatility-driven	liquid ↔ vapor	mature and robust, high purity, very high energy use, limited for heat-sensitive/azeotropic systems	bulk solvent recovery, solvent swap, volatile organic separation
adsorption	chemical potential gradient, surface affinity	low–medium for operation; medium–high for regeneration	surface adsorption and pore filling on solid adsorbents	no	highly selective for trace species, limited capacity, requires regeneration	color/odor removal, trace organic/metal removal, product polishing, dehydration
liquid Chromatography	pressure-driven flow, differential interaction with the stationary phase	low–medium (pumps, sometimes temperature control)	differential partition/adsorption between mobile and stationary phases	no	very high resolution, flexible selectivity, solvent-intensive, hard to scale up	high-purity API/fine-chemical purification, chiral separations, impurity profiling
evaporation	vapor pressure difference, heat supply	high (sensible + latent heat)	preferential evaporation of volatile component(s)	liquid → vapor	simple and robust, good for bulk concentration, low selectivity, thermal stress possible	solution concentration, partial solvent removal, preconcentration before crystallization
crystallization	supersaturation (chemical potential difference solution ↔ solid)	medium (cooling/heating, mixing)	nucleation and crystal growth from solution	liquid → solid	very high purity, enables polymorph control, sensitive to nucleation conditions	final API/fine-chemical isolation, solid–liquid separation from organic solvent mixtures

### 5.1 Purification

A primary application of OSN is purification, which separates two or more components through selective transport. This is particularly relevant to high-value manufacturing streams that require tight molecular separation. OSN is a pressure-driven, non-phase-change separation that can provide high throughput with relatively low energy demand compared with thermal methods.^116^ For purification, a key advantage of graphene-based membranes is the potential for a sharp MWCO transition, enabling high-purity separation when the product and impurity sizes are even close. Graphene-based OSN can offer a practical balance between resolution and scalability, bridging high-throughput bulk separations (e.g., evaporation/distillation) and high-resolution but scale-limited techniques (e.g., liquid chromatography).^21,53,117,118^ It can be particularly attractive when azeotropy or solvent/temperature constraints limit conventional thermal separations.^104^ Adsorption can complement graphene-based OSNs by removing trace species that are not efficiently captured by size-based sieving.^119^

Kang et al. developed the NG-200 membrane by employing a thermally induced pore tuning strategy on GO.^42^ In a 48-h continuous cross-flow filtration of a binary dye mixture of Methyl Red/Evans Blue (MR/EB), the NG-200 membrane demonstrated exceptional purification efficiency with a separation factor of approximately 1000. It maintained near-perfect rejection (∼100%) for EB (961 Da) while allowing MR (269 Da) to permeate with nearly 0% rejection. The robust selectivity and high-purity separation highlight its significant potential for precise filtration in industrial sectors.

### 5.2 Concentration

Beyond purification, concentration processes play a pivotal role in determining the final purity and yield of products. Increasing solute concentration facilitates downstream crystallization/precipitation and improves overall process efficiency.^1,120^ Conventional concentration by evaporation relies on phase change and typically entails high energy demand and potential thermal damage, whereas OSN-based concentration can be achieved without phase change via pressure-driven solvent removal.^121^ Graphene-based OSN membranes are particularly attractive because their ultrathin, laminar transport pathways can offer high permeance, enabling faster concentration compared with many conventional polymeric membranes.^122^ Properly stabilized graphene-based laminates can maintain a well-defined sieving window during extended concentration operation, even as viscosity and concentration polarization increase at higher solute loadings.^22^

Kim et al. developed a hybrid OSN membrane composed of CNTs and graphene nanoribbons (GNRs) to enable efficient purification and separation of organic mixtures.^39^ They successfully separated EB and MO dyes in IPA and achieved a long-term stability of 1315 h under cross-flow operation. The concentration capability was further indicated by recovering 76% of the remaining EB powder from a concentrated solution after multiple filtration cycles. These results demonstrate that graphene-based OSNs can sustain concentration and purification over extended operation.

### 5.3 Solvent Exchange and Recovery

The transition toward continuous-flow manufacturing in the fine chemical and pharmaceutical industries has accelerated due to enhanced productivity, reduced waste, and improved process control. A major bottleneck in such continuous systems remains the complexity of downstream separation, as multistep syntheses often require frequent solvent exchange between reaction conditions.^123^ Solvent management in multistep organic processes typically relies on a combination of distillation and liquid–liquid extraction.^119^ Distillation is a mature option for solvent exchange, but it is energy-intensive and can be limited for heat-sensitive streams or azeotropic systems.^124,125^ Solvent extraction is effective when immiscible phases exist and partitioning is favorable.^124–126^ OSN provides a pressure-driven, non-phase-change route for solvent exchange, polishing, and recycling even in fully miscible organic systems. Graphene-based OSN membranes are particularly attractive because their laminate structure can be engineered to provide solvent-tolerant operation and a well-defined sieving window for diverse organic solvents, reducing performance drift associated with solvent-induced swelling in many polymeric systems.^127^ In practice, OSN can function as a modular unit operation for solvent management (e.g., solvent exchange, solvent polishing, and recycle) in multistep workflows.

Kang et al. developed a microwave-treated nanoporous graphene (MNG) membrane platform designed for high-resolution ternary mixture separation.^22^ By exploiting the solvent-dependent “switching” of the membrane’s MWCO, the researchers demonstrated a sequential purification process that transitions through IPA, EtOH, and NMP, enabling the precise, stage-wise separation of multiple solutes within a single integrated system. Accordingly, solvent sequences and transition conditions should be designed in combination with the target molecular-weight window, so that shifts in the sieving window are treated as an explicit process design variable. This solvent-responsive concept reduces the need for membrane replacement between stages and supports integrated, multisolvent systems.

## 6 Future Perspectives for Graphene-Based Organic Solvent Nanofiltration Membranes

Graphene-based membranes have demonstrated outstanding OSN performance in numerous laboratory studies, yet their practical industrial deployment remains limited. To clarify the key requirements and bottlenecks for real-world implementation, we provide an integrated roadmap of performance, stability, module design, and industrial implementation (Figure 11). Then, we discuss future directions and high-value application opportunities of the graphene-based membrane.

**Figure 11:**
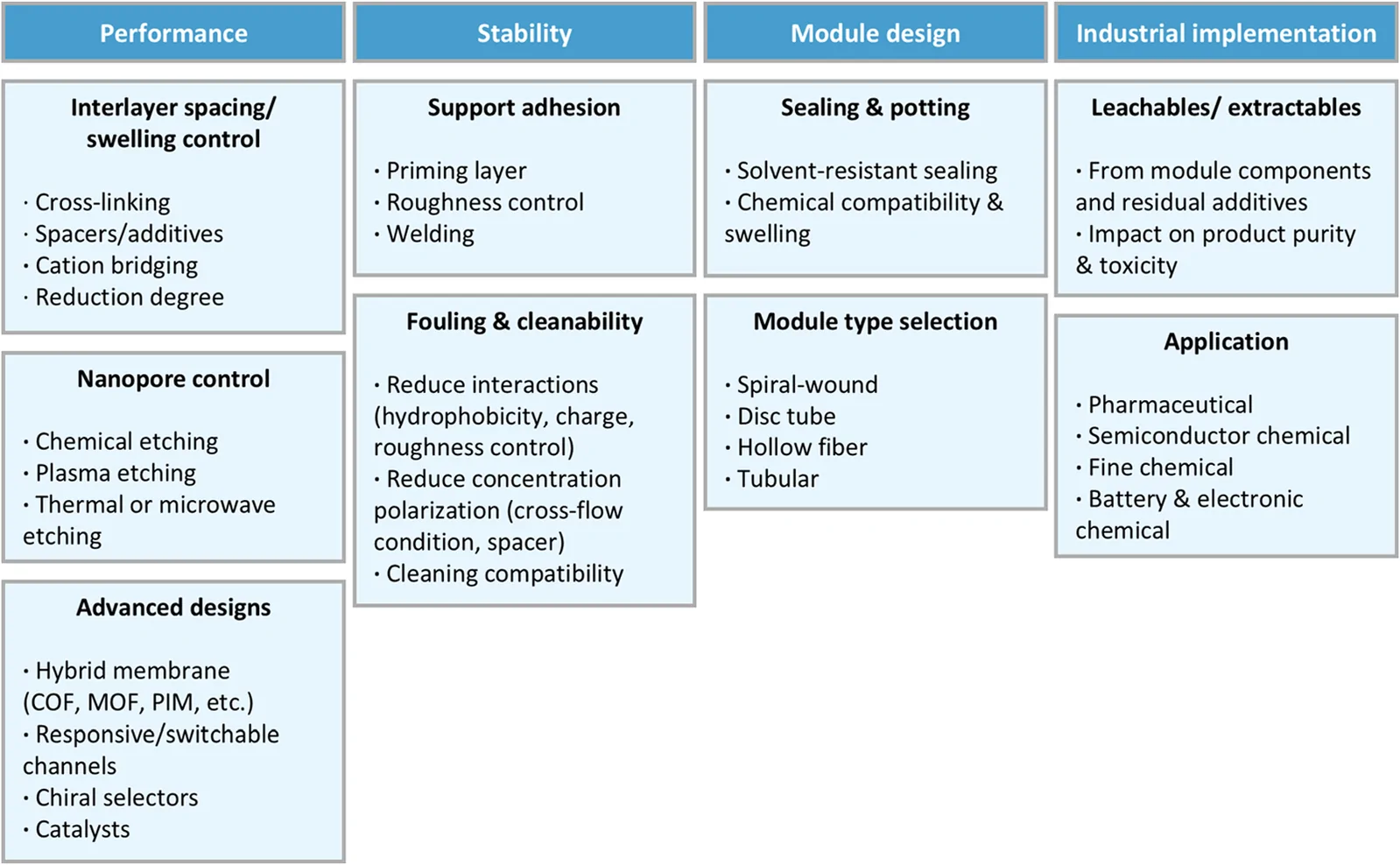
Key research directions and industrial considerations for advancing graphene-based membranes in organic solvent nanofiltration.

### 6.1 Scale-Up and Industrialization

As shown in Figure 12A, commercializing GO-based OSN membranes requires a transition from laboratory-scale fabrication to manufacturing processes that enable large-area, continuous coating. A practical bottleneck during scale-up is the trade-off between structural tuning and processability.^103^ Strategies used to regulate interlayer spacing and pore structure, such as reduction, cross-linking, and functionalization, often change solution stability, viscosity–concentration window, dispersibility, and coating/drying behavior, making large-area uniform deposition more challenging.^128,129^ To mitigate this limitation, a post-treatment manufacturing route has been demonstrated in which large-area GO coatings are first deposited from a well-dispersed solution, and the GO structure is subsequently tuned in the coated membrane (Figure 12B,C).^21^ For instance, combining scalable slot-die coating with hot-press post-treatment enables simultaneous regulation of interlayer and nanopore structure while strengthening the support-selective-layer stability within a single manufacturing route.

**Figure 12:**
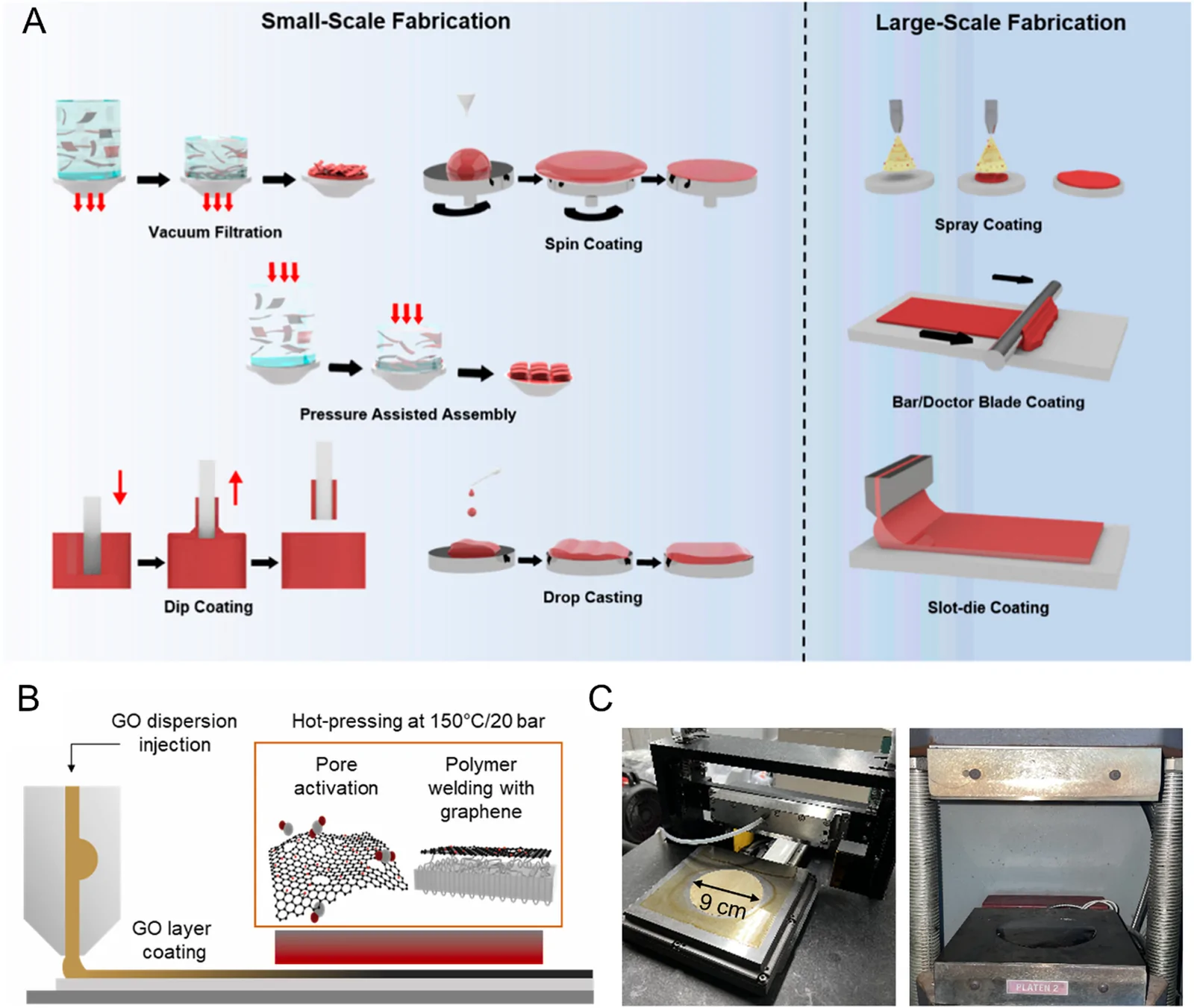
(A) Overview of fabrication methods for graphene-based membranes categorized by scalability. Reprinted with permission under a Creative Commons CC BY 4.0 from ref 103. Copyright 2021 MDPI. (B) Large-scale fabrication of nanoporous graphene oxide membrane combining slot-die coater and hot-press, and (C) photographs of processes. Reprinted with permission from ref 21. Copyright 2023 Elsevier.

Moreover, scale-up extends beyond area enlargement and demands manufacturing–integration solutions that include packaging into module systems.^130^ In OSN, these challenges are further amplified compared with water treatment because not only the membrane itself, but also the entire module hardware, including the housing, spacers, potting/sealing materials, and adhesive layers, is exposed to organic solvents over long periods.^131,132^ As a result, solvent-resistant module components and overall chemical compatibility are simultaneously required. Accordingly, industrialization of graphene-based membranes requires both packaging the membranes into module types suitable for the membrane and developing complete module hardware capable of stable operation under long-term exposure to organic solvents.

### 6.2 Organic Solvent Reverse Osmosis

The precise transport mechanism is fundamental to designing and extending high-performance GO-based membranes for organic solvent reverse osmosis (OSRO). Historically, the transport of fluids through dense or nanoporous membranes has been interpreted through two distinct theoretical frameworks: the Pore-Flow (PF) model (Figure 13A) and the Solution-Diffusion (SD) model (Figure 13B).^133,134^ The SD model assumes that permeants dissolve into the membrane matrix and diffuse through it down a chemical potential gradient, governed by Fickian diffusion. In contrast, the PF model describes transport as pressure-driven convection through continuous pores, typically governed by Darcy’s law, where separation arises primarily from steric exclusion based on size.^135^ Recent studies have suggested that water can be transported predominantly by pore flow through transient network defects, even in RO membranes.^135^ However, unlike cross-linked polymer networks, graphene-based laminar membranes exhibit a unique, pressure-dependent structural evolution, necessitating a dynamic interpretation of their transport behavior relative to the operating regime.^136,137^

**Figure 13:**
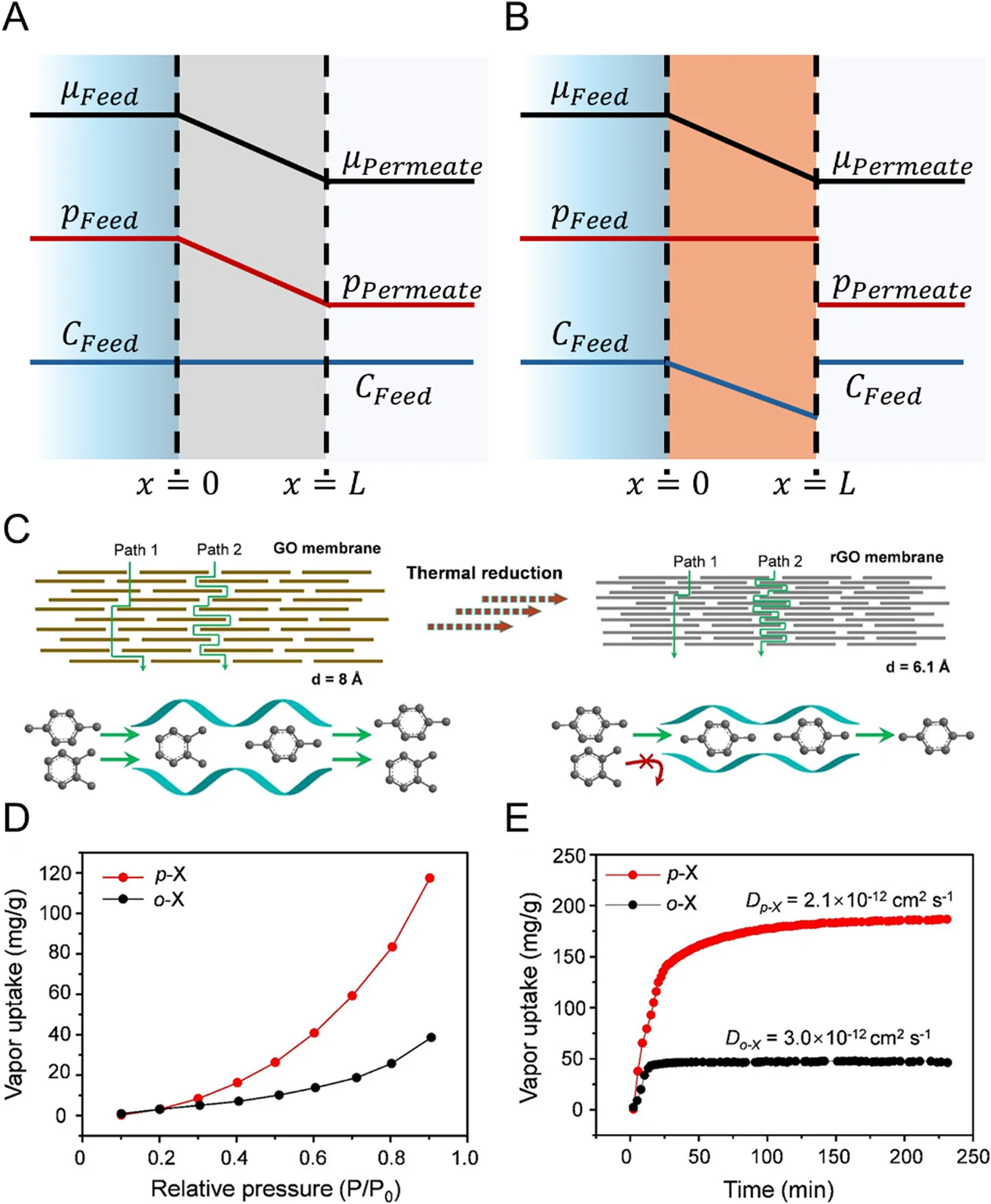
Comparison of transport mechanisms and a representative case study of membranes for OSRO applications. Schematics illustrating (A) the pore-flow model (OSN) and (B) the solution-diffusion model (OSRO) with the corresponding profiles of chemical potential (μ), solvent concentration (C), and pressure (p) across the membrane. (C), Structural evolution of GO and rGO laminates for xylene isomer separation. (D–E) Sorption isotherms and kinetic uptake profiles of *ortho*-xylene (*o*-X) and *para*-xylene (*p*-X) in the rGO membrane, demonstrating solubility and diffusivity selectivity. Reprinted with permission from ref 137. Copyright 2024 American Chemical Society.

In the regime of OSN, which typically operates at lower hydraulic pressures, graphene oxide laminates often retain a swollen structure due to solvent intercalation.^75^ In this state, the expanded interlayer channels allow for convective solvent transport, typically fitting the description of the PF model coupled with steric hindrance.^12^ However, the scenario could be changed under the rigorous conditions of the OSRO, where high applied pressure becomes a critical variable. Subjected to these high pressures, graphene laminates undergo significant structural compaction.^136^ This physical compression overcomes the solvation forces between the sheets, drastically reducing the interlayer spacing (d-spacing) and eliminating the free volume required for bulk-like convective flow. As the graphene sheets become rigidly locked, the membrane effectively transforms into a dense film. Transport may be more appropriately rationalized within an SD model influenced by thermodynamic solubility and kinetic diffusivity of the penetrating molecules.^136,137^

A representative study by Alemayehu et al. supports this interpretation for high-performance OSRO applications.^137^ As illustrated in Figure 13C, the authors utilized rGO membranes with precisely tuned interlayer spacing to challenge the difficult separation of xylene isomers (*p*-xylene vs o-xylene). The authors analyzed the transport behavior through sorption isotherms (Figure 13D) and kinetic uptake profiles (Figure 13E). By mathematically decoupling the permeability coefficient into its constituent terms-solubility (*S*) and diffusivity (*D*)-via the relation *P* = *S* × *D*, they demonstrated that the superior separation performance was driven by diffusivity selectivity. This finding indicates that within the tightly compacted graphene channels characteristic of OSRO applications, the transport is an activated diffusion process. When the GO laminate structure is stabilized under high pressure and interlayer transport pathways are precisely controlled, GO-based membranes could potentially be applied to OSRO-relevant separations, such as aromatic/isomer separations and polar–nonpolar fractionation.^138^

### 6.3 Integration into Pharmaceutical and Fine Chemical Processes

Pharmaceutical and fine chemical manufacturing are among the most promising near-term applications of graphene-based OSNs. In the pharmaceutical industry, OSN can be applied directly onto core unit operations in drug synthesis, including active pharmaceutical ingredient (API) concentration, impurity purge (e.g., diafiltration), catalyst retention/recovery, and solvent exchange across multistep routes (Table 4).^78^ In contrast, fine-chemical production often involves greater variability in solvents and compositions, so OSN is applied more widely to general-purpose operations such as catalyst recovery and reaction cleanup, product/byproduct separation, and solvent management.^139,140^ Graphene-based membranes are attractive because their high permeance and broad solvent compatibility can shorten cycle times and facilitate membrane-enabled unit operations within continuous manufacturing. Given the broad solvent stability of graphene-based membranes, synthesis processes can be designed with the most effective solvents for reaction or crystallization, followed by efficient solvent recovery via OSN.^6,141^

**Table 4:** Summary of Key Applications and Technical Requirements for OSN in Major Industries

application	typical purpose	typical solvents	MWCO range (Da)	permeance/flux requirement	key requirements
pharmaceutical	API concentration and purification, Solvent swap	alcohols, ACN, DMF, DMSO, THF	tight OSN (∼200–1000 Da)	stable flux at high solids (10–40 wt %)	very high rejection of API/critical impurities, extremely low leachables/extractables, compatibility with multisolvent recipes, GMP/ICH Compliant
semiconductor chemicals	purification of ultrahigh-purity solvents, metal/ion removal	IPA, PGMEA, PGMEA, esters	tight NF/OSN (∼200–500 Da)	stable long-term operation, modest flux acceptable	ultralow metals/TOC
					no particle shedding
					UP/EL grade specifications;
fine chemicals	catalyst recovery, reaction cleanup, product/byproduct fractionation	wide range: alcohols, ketones, esters (EA), polar aprotics, hydrocarbons	broad OSN window (∼300–1000 Da)	high flux preferred, tolerance to variable feeds	strong solvent stability, good rejection of the target species, and handle changing compositions/temperatures
battery & electronic chemicals	recycling of battery solvents/binders, electrolyte purification	NMP, DMF, DMAc, carbonate solvents, ethers, IPA/EtOH for cleaning	UF (>10,000 Da) for binders, tight OSN (∼200–500 Da) for low-MW impurities	high flux for viscous slurries, moderate flux for polishing	high chemical stability with Li salts, low metal/ionic leaching, control of moisture uptake (for battery electrolytes);

In pharmaceutical translation, deployment is governed not only by membrane performance but also by stringent quality and regulatory requirements. Pharmaceutical OSN typically demands a tight MWCO window (≈200–1000 Da) and stable permeance under high-solid feeds (≈10–40 wt %), and, critically, it must suppress leachables/extractables to ultralow levels while meeting GMP/ICH-grade robustness.^1,142^ Graphene-based laminates are attractive because a sharp MWCO transition can enable high-purity separation. Recent reviews further indicate that graphene-based membranes can achieve >95% rejection for a range of pharmaceutical solutes in organic solvents, supporting the feasibility for product purification and enrichment.^143,144^ In addition, nanofiltration could replace distillation by removing a high-boiling solvent from a heat-sensitive API solution.^145^

Fine-chemical applications often require separations in the 300–1000 Da window for removing excess reagents, ligands/side-products, and homogeneous catalyst residues while handling frequent solvent swaps and solvent recycle across processes.^146^ Graphene-based OSN could provide a flexible platform for integrating continuous reactions with downstream purification and solvent management in a single workflow. Realizing this opportunity, however, will require membrane tuning that explicitly accounts for solvent-dependent swelling and interaction behavior, as well as reliability validation under industrially relevant cross-flow conditions.

### 6.4 Electronic-Grade Solvent Purification

Electronic and energy material value chains impose a distinct constraint set in which purity assurance and contamination control dominate the specification space. In semiconductor and electronic-grade solvent purification, ultrapure process solvents such as propylene glycol methyl ether acetate (PGMEA), propylene glycol methyl ether (PGME), NMP, and IPA are required, with impurity limits often specified at the ppb–ppt level.^147–149^ However, the substantial procurement cost of ultrapure solvents makes a “use-and-dispose” model economically impractical.^150^ Currently, these are purified by multistep distillation and expensive polishing steps.^149^ Graphene-based OSN membranes could be applied to remove trace contaminants (metal ions, oligomers, particles) from solvents used in microelectronics, with less energy than distillation.^10^ The high chemical inertness of graphene-based materials is crucial here, as even ppb-level leaching from a membrane could contaminate semiconductor processes.^151^ Unlike polymeric membranes, which often suffer from swelling-induced leaching in harsh solvents or adsorption of organics, graphene-based laminates, when engineered with robust cross-linking, maintain high structural integrity.^152–154^ This minimizes the risk of extractables or degradation products, which are critical for semiconductor photolithography and cleaning steps. Additionally, the inherent thermal stability of graphene-based materials enables solvent purification at elevated temperatures, further enhancing process efficiency in stripping or cleaning applications.^155^

Battery and electronic-chemical streams similarly prioritize solvent reuse and impurity control, including recycling of battery/binder-related solvents (e.g., NMP/DMF/dimethylacetamide (DMAc)) and purification of carbonate electrolyte solvents.^156^ The operating environment is more severe because of high viscosity and Li salt presence, requiring membranes that preserve performance while suppressing metal/ionic leaching and water uptake.^157,158^ Graphene-based membranes can be attractive for solvent reuse loops and impurity removal, but implementation depends on stability against salt-induced interactions, concentration polarization in viscous media, and durable module compatibility over long-term operation.

### 6.5 Advanced Membrane Designs

We anticipate more research on hybrid membranes that combine graphene with other emerging materials (e.g., COF, MOF, polymer of intrinsic microporosity (PIM) coatings). For example, Sui et al. reported 2D-COF-intercalated rGO laminates in which COFs act as nanospacers/stabilizers/porous fillers, mitigating swelling while creating transport shortcuts through intrinsic in-plane pores for higher OSN permeance.^159^ These also could synergistically improve selectivity for specific separations. For instance, a graphene membrane embedded with chiral pores (via a functional additive) could perform enantiomer separation of pharmaceuticals. In fact, preliminary studies have modified graphene oxide membranes with cyclodextrin-based chiral selectors and observed enhanced enantiomer discrimination.^160^ Similarly, we expect continued development of dual-function membranes that respond to stimuli. The solvent-switchable PG/GO membrane is one example; future membranes might respond to electric fields or pH to modulate pore size on demand.^78^ For OSN in multistep syntheses, a single “smart membrane” could perform a sequence of separations by adjusting an external stimulus, simplifying process design.

### 6.6 Catalytic Filter

Graphene-based OSN membranes can be extended into catalytic filters by integrating catalytic functionality either on the surface or within transport channels, thereby enabling simultaneous molecular sieving and catalytic reaction. Catalytic membranes in water treatment have been used primarily for foulant degradation and cleaning.^161,162^ From an OSN perspective, catalytic functionality can further expand the role of graphene-based membranes beyond separation and purification, toward reactive separation platforms that couple molecular sieving with in situ reaction^163^ under nanoconfinement. For example, Li et al. demonstrated a GO-based laminar “membrane flow-reactor” for aspirin synthesis, achieving nearly complete conversion under directional flow. This was attributed to the synergistic effect of subnanometer confinement and the tuned physicochemical/electronic structure of GO. Beyond such demonstrations, molecular sieving in nanoconfined graphene-based channels may allow codesign of reaction and separation by tuning both reaction selectivity and the sieving window within a single membrane platform. Nevertheless, several bottlenecks remain in organic-solvent environments, including catalyst/ligand leaching, channel blockage caused by the introduction of catalytic components, catalyst deactivation/regeneration, and stability.^164,165^ Therefore, future progress will require combining solvent-resistant immobilization chemistries with long-term operation to establish practical conditions that satisfy both catalytic function and OSN performance.^166^

### 6.7 Addressing Challenges

An issue in current graphene-based OSN research is the heavy reliance on rigid dye molecules as rejection probes.^167^ Although dyes enable straightforward UV–Vis detection, their separation behavior often differs from that of flexible industrial solutes due to charge effects, anisotropic geometry, and hydration/solvation shell differences.^168,169^ Consequently, the molecular weight of dyes does not consistently correlate with their effective hydrodynamic size in organic solvents (Figure 14). Testing should prioritize real industrial solutes, such as APIs and catalysts, to validate membrane performance under realistic process conditions where solute–solvent–membrane interactions are complex and dynamic.

**Figure 14:**
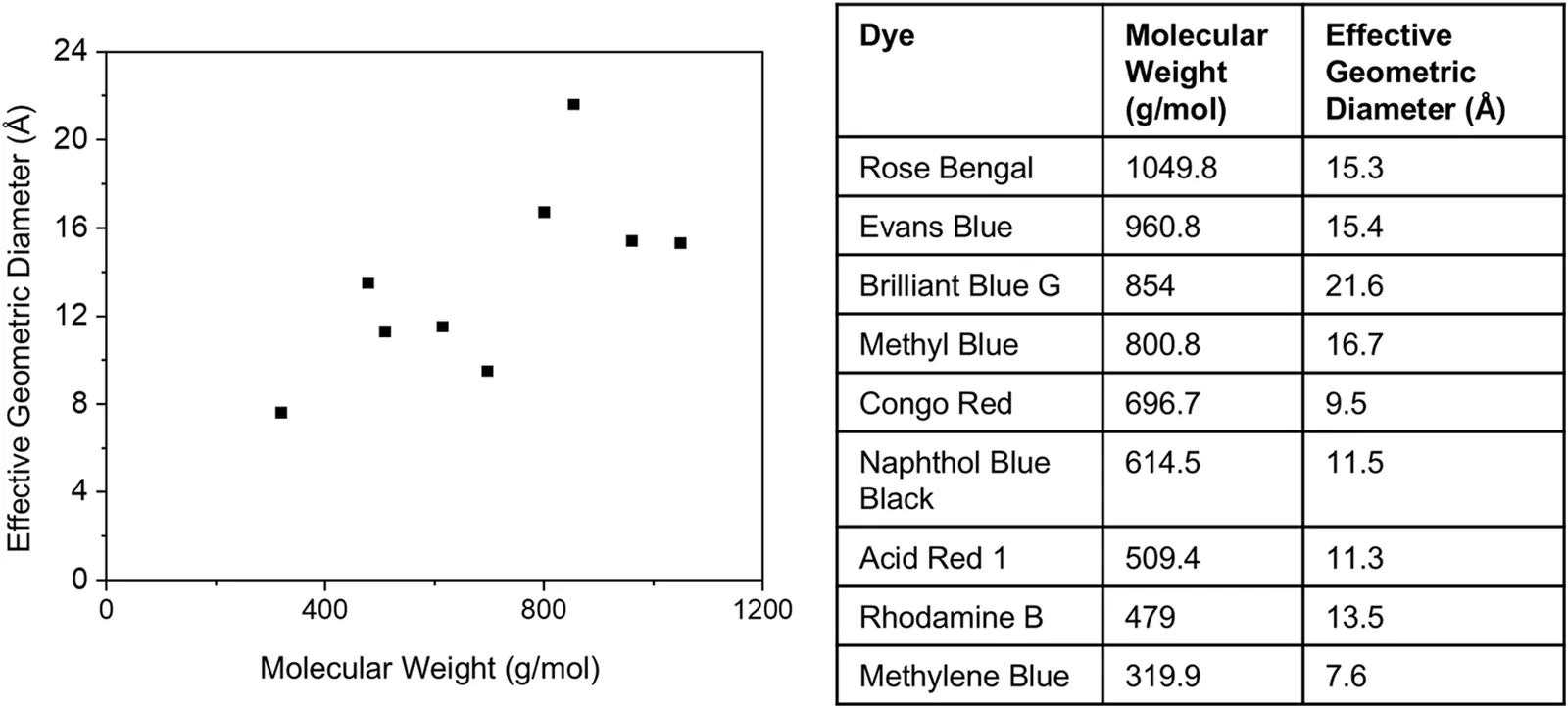
Relationship between molecular weight and effective geometric diameter of various organic dyes. The effective geometric diameter was defined by measuring the diameter of the minimum cross-section after rendering the van der Waals surfaces of dye molecules.

Despite the rapid progress, several practical challenges still hinder the translation of graphene-based membranes to robust OSN platforms. First, precise interlayer spacing control should be maintained under long-term solvent exposure. This requires solvent-resistant additives that stabilize the nanochannels without leaching and preserve long-term performance by suppressing compaction.^170^ In addition, pore-size homogeneity at scale is essential to ensure a consistent product cutoff. In parallel, continued advances in support engineering are required not only to develop supports that are chemically compatible with aggressive organic solvents but also to enhance adhesion at the graphene/support interface to prevent delamination.^171,172^ To address the intrinsic limitations of polymeric supports, researchers are exploring ceramic or metallic supports, as well as self-supporting graphene architectures such as carbon nanotube supports.^173,174^ Finally, fouling mitigation remains critical in realistic feeds, where adsorption of organics and process impurities can rapidly degrade flux and apparent selectivity.^167^ In graphene-based membranes, fouling can occur through multiple pathways, including π–π interactions between aromatic solutes and graphitic domains, hydrogen bonding or electrostatic interactions with oxygen-containing groups, accumulation on the surface and pore or interlayer-channel blockage by retained molecules.^175^ These effects are particularly critical in high-concentration or high-viscosity feeds, where concentration polarization can increase the local solute concentration near the membrane surface, leading to flux decline or apparent changes in rejection.^176^ Therefore, practical implementation requires both antifouling-oriented surface design and process-level fouling control, such as reducing adsorption through surface functionalization, optimizing cross-flow velocity and spacer geometry, and applying periodic solvent flushing or chemical cleaning protocols.^177,178^ Addressing these coupled issues will be essential to move from compelling lab-scale demonstrations to commercially viable OSN modules.

## 7 Conclusions

Across recent studies, performance improvements of graphene-based membranes have been driven not only by material composition but by precise structural engineering strategies, including nanopore formation and sealing, interlayer spacing regulation, nanosheet orientation control, and building-block geometry design. These approaches collectively demonstrate that the balance between permeance and selectivity can be fundamentally redefined by controlling nanochannel structure and transport pathways. Multilayer graphene-based membranes exhibit hybrid transport behavior governed by the interplay between pore-flow transport, interlayer confinement, and solvent–membrane interactions. Importantly, solvent-induced structural dynamics and interfacial slip effects play decisive roles in determining real OSN performance, emphasizing the need for solvent-specific design strategies.

Despite remarkable laboratory achievements, several critical barriers remain for the industrial implementation. Long-term structural stability under aggressive organic solvents, scalable manufacturing routes, module-level integration, and realistic testing using industrial solutes rather than model dyes are essential next steps. Furthermore, the emerging transition from OSN toward organic solvent reverse osmosis highlights the importance of understanding the pressure-dependent structural evolution and transport mechanisms. Future research directions are expected to arise from hybrid architectures, responsive or multifunctional membranes, and catalytic separation platforms that integrate reactions and filtration within nanoconfined environments. By bridging fundamental nanochannel engineering with process-oriented design and manufacturing considerations, graphene-based membranes are expected to be key technologies for sustainable chemical processing, pharmaceutical manufacturing, and advanced solvent-management systems.
